# A Metabologenomic Approach Reveals Changes in the Intestinal Environment of Mice Fed on American Diet

**DOI:** 10.3390/ijms19124079

**Published:** 2018-12-17

**Authors:** Chiharu Ishii, Yumiko Nakanishi, Shinnosuke Murakami, Ryoko Nozu, Masami Ueno, Kyoji Hioki, Wanping Aw, Akiyoshi Hirayama, Tomoyoshi Soga, Mamoru Ito, Masaru Tomita, Shinji Fukuda

**Affiliations:** 1 Institute for Advanced Biosciences, Keio University, 246-2 Mizukami, Kakuganji, Tsuruoka, Yamagata 997-0052, Japan; chiharu@sfc.keio.ac.jp (C.I.); yumiko.sato.kj@riken.jp (Y.N.); mushin@sfc.keio.ac.jp (S.M.); wanping@sfc.keio.ac.jp (W.A.); hirayama@ttck.keio.ac.jp (A.H.); soga@sfc.keio.ac.jp (T.S.); mt@sfc.keio.ac.jp (M.T.); 2 Systems Biology Program, Graduate School of Media and Governance, Keio University, 5322 Endo, Fujisawa, Kanagawa 252-0882, Japan; 3 Central Institute for Experimental Animals, 3-25-12 Tonomachi, Kawasaki-ku, Kawasaki, Kanagawa 210-0821, Japan; rnozu@ciea.or.jp (R.N.); mueno@ciea.or.jp (M.U.); hioki@ciea.or.jp (K.H.); mito@ciea.or.jp (M.I.); 4 Department of Environment and Information Studies, Keio University, 5322 Endo, Fujisawa, Kanagawa 252-0882, Japan; 5 Intestinal Microbiota Project, Kanagawa Institute of Industrial Science and Technology, 3-25-13 Tonomachi, Kawasaki-ku, Kawasaki, Kanagawa 210-0821, Japan; 6 Transborder Medical Research Center, University of Tsukuba, 1-1-1 Tennodai, Tsukuba, Ibaraki 305-8575, Japan; 7 PRESTO, Japan Science and Technology Agency, 4-1-8 Honcho Kawaguchi, Saitama 332-0012, Japan

**Keywords:** metabologenomics, microbiome, metabolome, intestinal microbiota, next-generation sequencing, CE-TOFMS, multi-omics, American diet

## Abstract

Intestinal microbiota and their metabolites are strongly associated with host physiology. Developments in DNA sequencing and mass spectrometry technologies have allowed us to obtain additional data that enhance our understanding of the interactions among microbiota, metabolites, and the host. However, the strategies used to analyze these datasets are not yet well developed. Here, we describe an original analytical strategy, metabologenomics, consisting of an integrated analysis of mass spectrometry-based metabolome data and high-throughput-sequencing-based microbiome data. Using this approach, we compared data obtained from C57BL/6J mice fed an American diet (AD), which contained higher amounts of fat and fiber, to those from mice fed control rodent diet. The feces of the AD mice contained higher amounts of butyrate and propionate, and higher relative abundances of *Oscillospira* and *Ruminococcus.* The amount of butyrate positively correlated with the abundance of these bacterial genera. Furthermore, integrated analysis of the metabolome data and the predicted metagenomic data from Phylogenetic Investigation of Communities by Reconstruction of Unobserved States (PICRUSt) indicated that the abundance of genes associated with butyrate metabolism positively correlated with butyrate amounts. Thus, our metabologenomic approach is expected to provide new insights and understanding of intestinal metabolic dynamics in complex microbial ecosystems.

## 1. Introduction

Intestinal microbiota, which consists of a large number of bacteria, archaea, viruses, and fungi, inhabit the gastrointestinal tracts of animals, including humans [1]. These microbial communities have been shown to contribute to food digestion, nutrient absorption, and development of the host immune system. Recent rapid advances in DNA sequencing technology have allowed researchers to conduct in-depth studies of the structures of intestinal microbiota and their effects on host physiology. Previous studies have reported that intestinal microbial dysbiosis could be a risk factor for several diseases, including colon cancer [2,3], hepatic cancer [4], obesity [5,6,7], diabetes [8,9,10], atherosclerosis [11,12,13], immune system disorders [14,15], and brain function disorders [16,17,18,19,20]. 

On the other hand, recent studies have found that intestinal microbiota produces a range of low-molecular-weight metabolites such as short-chain fatty acids (SCFAs) [21,22], trimethylamine [12], indole metabolites (e.g., indole propionate, indole-3-acetaldehyde) [23,24], vitamins [25,26], polyamines [27,28], and secondary bile acids [29]. These molecules play direct and/or indirect roles in maintaining good health and suppressing various diseases. For example, intestinal microbiota-produced acetate that is secreted into the systemic circulation after absorption from the intestinal lumen has been shown to suppress asthma [15]. Additionally, it has been reported that acetate produced by probiotic *Bifidobacterium* improves intestinal defense and protects the host from enteropathogenic infection [30]. In another case, acetate-mediated activation of G-protein-coupled receptor (GPCR) 43 suppresses insulin signaling in adipocytes, and regulates energy balance by suppressing accumulation of excess energy and promoting fat consumption [31]. Furthermore, butyrate, one of the most abundant SCFAs produced by intestinal microbiota, induces the differentiation of naïve T cells to colonic regulatory T cells; these T_reg_ cells play a pivotal role in suppressing inflammatory and allergic responses, thereby attenuating colonic inflammation in mice [32]. These studies indicate that intestinal microbiota-derived metabolites are as important as the composition of intestinal microbiota; characterization of these metabolites is needed to fully understand the relationship between the intestinal ecosystem and human health.

We therefore inferred that we could obtain novel knowledge by combining the metabolome and microbiome analysis of the intestinal environment. A multi-omics approach could be a valuable tool for understanding the entire intestinal ecosystem, including the relationships among microbiota, metabolites, and host. Additionally, it is considered that a multi-omics approach can provide new knowledge about the mechanisms underlying the key roles the microbiota plays in the varying conditions of the intestinal environment. To gain new insight and knowledge from omics data, the data analysis process is highly important. For example, there are some analytical methods or pipelines for analyzing DNA sequence datasets obtained from microbiota. Quantitative Insights into Microbial Ecology (QIIME) [33] and mothur [34] are used for processing raw sequence datasets to microbiome profiles and comparing these profiles. Linear Discriminant Analysis (LDA) Effect Size (LEfSe) [35] is used for finding factors that explain the differences between microbial communities. Phylogenetic Investigation of Communities by Reconstruction of Unobserved States (PICRUSt) [36] is used for predicting the function of the microbial communities from 16S rRNA encoding gene sequence dataset. Analytical methods or pipelines for metabolome dataset such as MetaboAnalyst [37] and MetaCore (GeneGo, Inc.) pick up factors that might explain the differences between sample groups. However, analytical pipelines for multi-omics dataset are not well developed. Therefore, it is necessary to combine existing methods, or develop original methods to comprehensively analyze the relationships between the intestinal microbiome and metabolome. Complicated computational analysis is a potential barrier for scientists utilizing a multi-omics approach in this field. For that reason, although there have been studies that investigated microbial structure and/or intestinal metabolome profiles [38,39,40], there have been only a limited number of studies that performed a comprehensive analysis of multi-omics datasets using correlation and network analysis [41,42]. Therefore, to obtain novel information and further clarify entire intestinal microbial ecosystems, it is highly important to develop easy-to-use analytical systems for multi-omics datasets.

Here, we describe our original analytical strategy, metabologenomics, an integrated analysis of mass spectrometry-based metabolome data and high-throughput-sequencing-based microbiome data that seeks to understand the intestinal ecosystem. In the present study, we utilized the metabologenomic approach to clarify the relationships between the intestinal metabolome and microbiome profiles of mice fed control or American diet (AD). Our metabologenomic approach can detect detailed information regarding changes to the profiles of the intestinal metabolome and microbiome, and regarding the interactions among metabolite concentrations, microbiome abundance, and microbiome gene sets in the gut of mice maintained on AD. This approach provides new insights, facilitating our understanding of intestinal metabolic dynamics in complex microbial ecosystems.

## 2. Results

### 2.1. Development of the Metabologenomic Approach

**Figure 1:**
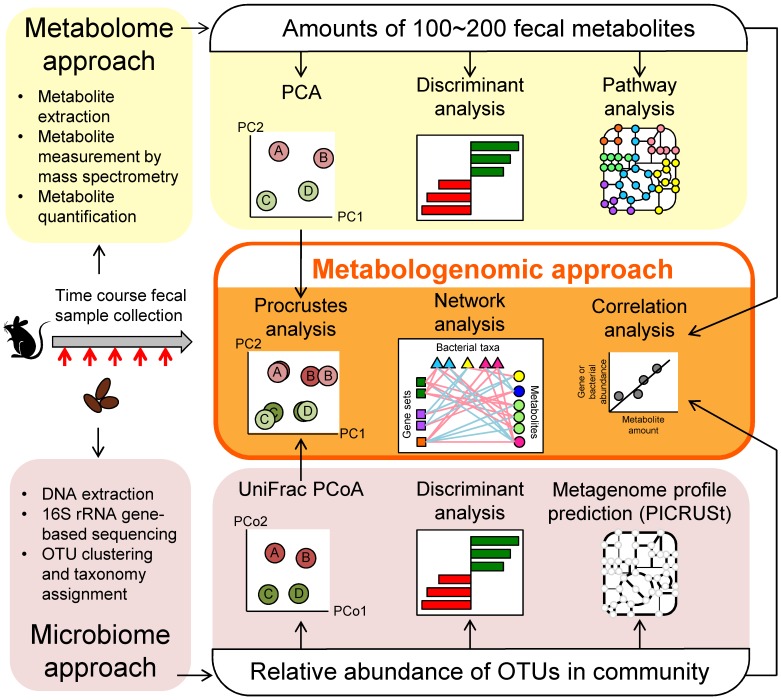
Overview of metabologenomic analysis workflow. Steps used for evaluation of metabolome profiles are summarized in the top row (yellow shading). This process starts with measurement of the amount of fecal metabolites to obtain profiles for the 100–200 metabolites. These metabolome profiles then are compared using Principal Component Analysis (PCA), discriminant analysis, and pathway analysis. Steps used for evaluation of microbiome profiles are summarized in the bottom row (pink shading). This process starts with the sequencing of the community’s 16S rRNA-encoding genes to clarify the relative abundance of operational taxonomic units (OTUs). Microbial memberships and structures are compared using UniFrac principal coordinate analysis (PCoA) and discriminant analysis. Additionally, Phylogenetic Investigation of Communities by Reconstruction of Unobserved States (PICRUSt) is used to predict metagenomic profiles. Steps for metabologenomic analysis are summarized in the central part of the figure (middle row; orange shading). The PCoA and/or PCA plots are used for Procrustes analyses. The relative abundances of microbial taxonomy and/or metagenome profiles and amounts of metabolites then are used for correlation analysis and network analysis.

First, to obtain novel information regarding the whole intestinal microbial ecosystem, we designed an original approach, Metabologenomics, for analysis of the multi-omics dataset. This system consists of separate analyses of the intestinal metabolome and microbiome, as well as an integrated analysis of the combined intestinal metabolome and microbiome datasets. An overview of the study design is depicted in Figure 1; details of these computational analyses are presented schematically in Figure S1. For this first example of the use of our metabologenomic approach, we obtained fecal samples from mice maintained on a control diet or AD, and these specimens were used for the extraction of metabolites and DNA. 

For the metabolome approach, amounts and/or relative abundances of metabolites could be measured comprehensively using mass spectrometry and/or NMR. In the present study, Capillary Electrophoresis Time-Of-Flight Mass Spectrometry (CE-TOFMS) was used to obtain the metabolome dataset. CE-TOFMS is a tool that can measure polar and ionic low-weight metabolites. It has been reported that CE-TOFMS can detect 179 metabolites from mice feces [43], and 214 fecal metabolites from school-aged children [44]; the detected compounds include SCFAs, amino acids, some vitamins, and polyamines. To clarify the differences in metabolites between the control and AD groups, principal component analysis (PCA), discriminant analysis, and statistical analysis were used. Additionally, Metabolite Set Enrichment Analysis (MSEA) [37] was used to evaluate pathways that differed between the two dietary groups. 

For microbiome analysis, V1–V2 regions of 16S rRNA-encoding genes were sequenced by MiSeq (Illumina). Sequence reads that passed the quality filters were clustered into Operational Taxonomic Units (OTUs) based on a cut-off of 97% similarity and assigned to the taxonomy using QIIME. To clarify the differences in microbiota components between the control and AD groups, UniFrac principal coordinate analysis (PCoA), discriminant analysis, and statistical analysis were used. Furthermore, to consider not only the microbiome structure but also the microbiome function, we utilized PICRUSt to predict the metagenome profiles based on 16S rRNA gene sequence data. To demonstrate the relationships between the intestinal metabolome and microbiome, Procrustes analyses were used to visualize and compare the two datasets. We conducted correlation analyses to obtain detailed information about relationships among the metabolome, microbiome, and predicted metagenome profiles. To simplify the complex interactions between metabolites and microbes, hierarchical clustering of autocorrelation maps was used to identify clusters that share the same patterns of changes, and hundreds of significant correlations were visualized in a network graph.

### 2.2. AD Consumption Alters Intestinal Metabolome Profiles

Fecal samples were obtained from male C57BL/6J mice fed control diet or AD (control diet: *n* = 6, AD: *n* = 5) from weaning at 8, 12, 24, 36, and 52 weeks (Figure S2A). Previous studies have reported that consumption of Western diets or food containing high concentrations of fat and/or carbohydrate can lead to intestinal microbial imbalance and induce diseases like colon cancer [2,3], hepatic cancer [4], obesity [5,6,7], diabetes [8,9,10], and atherosclerosis [11,12,13]. 

In this study, we used a laboratory chow formulated to match the daily human nutritional content in the United States [45]; the nutritional contents of AD and the control chow are summarized in Table S1. AD contains 15.5% fat and 5.3% fiber, whereas the control diet contains 5.0% fat and 3.5% fiber. AD represents a relatively moderate-fat diet compared to the other high-fat diets (which can contain 30–35% fat) that have been used as in previous studies [46,47,48].

**Figure 2:**
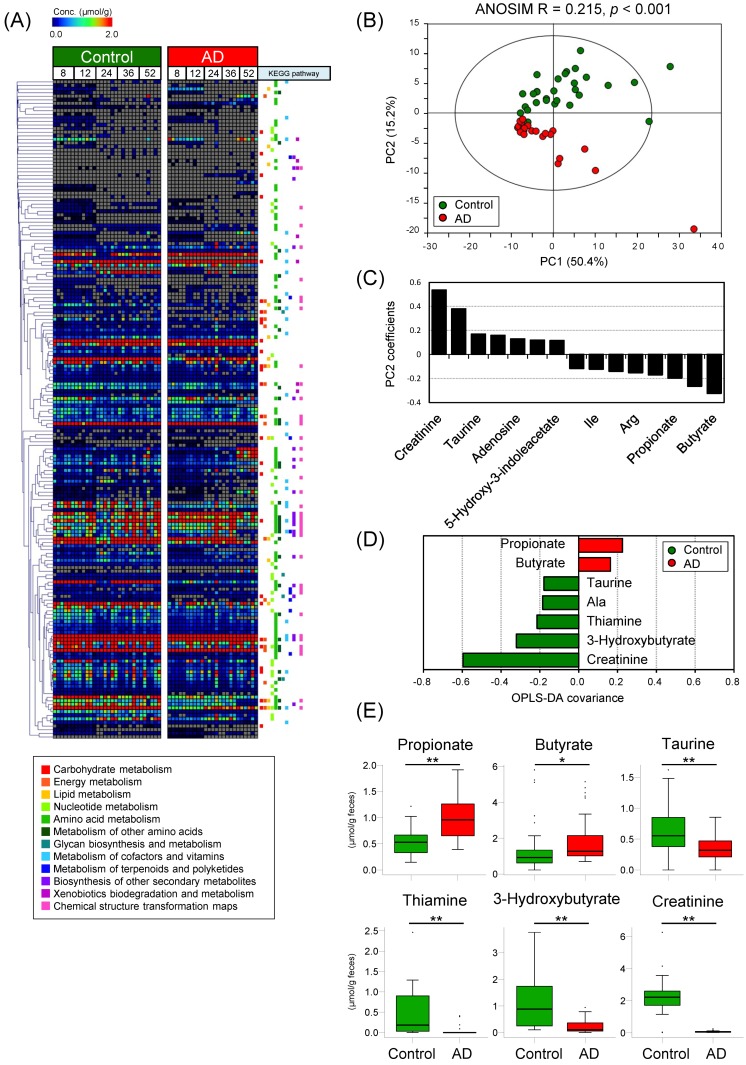
AD consumption alters intestinal metabolome profiles in mouse. (**A**) Heatmap showing the concentrations of quantified metabolites using a rainbow scheme. Gray indicates the concentrations of metabolites that fell below the detection limit. Kyoto Encyclopedia of Genes and Genomes (KEGG) pathways that each metabolite belongs to are shown to the right of the heatmap. Labels at the top of the panel indicate dietary group and mouse age (in weeks); (**B**) PCA of the intestinal metabolome profiles normalized by Pareto and analysis of similarity (ANOSIM). The ellipse denotes the 95% significance limit of the model, as defined by Hotelling’s *t*-test; (**C**) Bar graph showing PC2 values for metabolites that had |PC2 coefficients| > 0.11 in loading of PCA; (**D**) Bar graph showing OPLS-DA covariance values for metabolites that had |OPLS-DA covariance| > 0.16 based on OPLS-DA of metabolome profiles of control and AD mice. The model resulted in one predictive and one orthogonal four components with the cross-validated predictive ability Q2 (cum) = 0.863 and the total explained variance R2X (cum) = 0.791; (**E**) Box plots indicating fecal amounts of metabolites that had |PC2 coefficient values| > 0.11 in PCA, |OPLS-DA covariance values| > 0.16 in OPLS-DA, and false discovery rate (FDR) < 0.05 based on Mann–Whitney *U* test and Benjamini-Hochberg correction when comparing between the control and AD groups. Significant differences are indicated by * FDR < 0.05, ** FDR < 0.01.

There were no significant differences in body weights between control and AD mice (Figure S2B). A total of 184 fecal metabolites were detected by CE-TOFMS. These metabolites corresponded to various pathways, including the metabolism of carbohydrates, energy, lipids, and amino acids (Figure 2A). Of these 184 fecal metabolites, 84 metabolites were significantly different between the control and AD groups, as assessed by the Mann-Whitney *U* test (Table S2). Of these 84 metabolites, 74 showed decreased levels in the AD group (Table S2). To evaluate pathways that are involved in the 84 metabolites with significantly changed, MSEA was conducted. MSEA calculates whether a specific pathway is over-represented by chance within an arbitrary list of metabolites. MSEA showed that metabolites related to methionine metabolism were significantly changed between the control and AD groups (Table 1). To investigate whether metabolites contributed to the differences between the control and AD groups, multivariate analyses were conducted. PCA plots and analysis of similarities (ANOSIM) showed that the fecal metabolome profiles clustered into 2 groups depending on host diet (Figure 2B). However, the Euclidean distances were not significantly different based on host age or individual variability (Figure S3). According to the PC2 coefficients of the PCA, the amounts of butyrate, propionate, and amino acids such as Asp, Glu, Arg, Leu, Ile, and Met were higher, and the amounts of creatinine, 3-hydroxybutyrate, taurine, thiamine, and nucleosides were lower, in the AD group (Figure 2C). Additionally, orthogonal partial least squares discriminate analysis (OPLS-DA) showed results similar to those of PCA. According to the OPLS-DA covariance scores, higher amounts of butyrate and propionate, and lower concentrations of creatinine, 3-hydroxybutyrate, thiamine, Ala, and taurine in AD contributed to the separation of the two groups (Figure 2D). The metabolites for which the absolute amount values exceeded the threshold in both PC2 coefficients and OPLS-DA covariances are shown in the box plots in Figure 2E.

### 2.3. AD Consumption Alters Intestinal Metagenome Profiles

**Figure 3:**
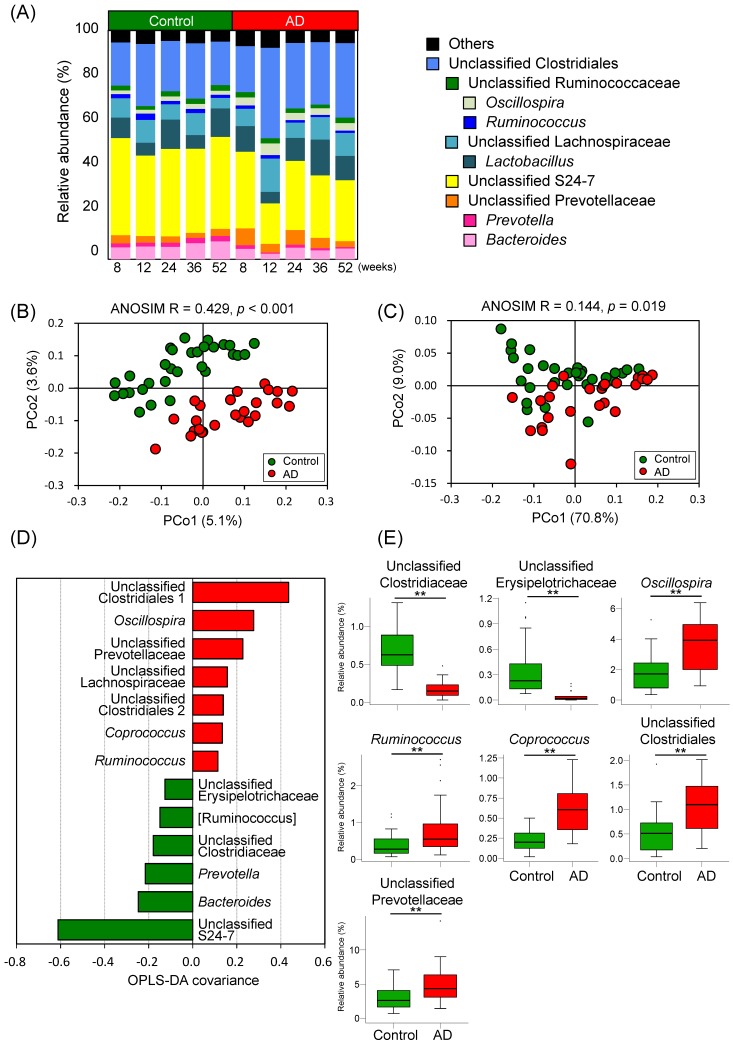
AD consumption alters intestinal microbiome profiles. (**A**) Bar graph showing the relative abundance of top 10 most-abundant genera (average abundance in all samples >1%) in control and AD mice; (**B**) Unweighted and (**C**) weighted UniFrac PCoA and ANOSIM comparing the intestinal microbiome profiles of control and AD mice; (**D**) Bar graph showing bacterial genera that had |OPLS-DA covariance| > 0.11 based on OPLS-DA of the microbiome profiles of control and AD mice. The model resulted in one predictive and one orthogonal four components with the cross-validated predictive ability Q2 (cum) = 0.832 and the total explained variance R2X (cum) = 0.918; (**E**) Box plots indicating relative abundance of genera that have |LDA score| > 2.0, |OPLS-DA covariance| > 0.11 in OPLS-DA, and FDR < 0.05 based on Mann–Whitney *U* test and Benjamini-Hochberg correction between the control and AD groups. Significant differences are indicated by * FDR < 0.05, ** FDR < 0.01.

To evaluate the impact of AD consumption on intestinal microbial composition, 16S rRNA-encoding genes were sequenced by MiSeq. A total of 880,770 reads of filter-passed 16S rRNA gene sequences were clustered into 1666 OTUs based on a minimum similarity of 97%. Genus-level microbial structures are shown as a bar graph in Figure 3A. Of 106 genera, 15 differed significantly in abundance between the control and AD groups (Table 2). Unweighted and weighted UniFrac principal coordinate analyses (PCoAs) and ANOSIM were conducted to compare the microbial membership and structure. The results of UniFrac PCoA and ANOSIM indicated the separation between control and AD in both unweighted and weighted analyses (Figure 3B,C). The distances between samples within the same dietary group were significantly shorter than the distances between different dietary groups, based on both unweighted and weighted UniFrac distances (Figure S4A). However, there was no significant difference in the distances between samples within the same age and of different ages, and samples within the same subject and of different subjects (Figure S4B,C). These results indicated that dietary condition has a bigger impact on intestinal microbial membership and structure than host age and individual variability. To identify the bacterial taxa that may contribute to this separation, OPLS-DA was performed [49,50,51]. The bacterial taxa that contribute to the separation are shown in Figure 3D. Additionally, as another method of discriminant analysis, we also conducted LEfSe analysis [35]. LEfSe results showed that 39 taxa contributed to the separation between the control and AD groups (Figure S5A). The taxa that were present at higher proportions in the AD group were distributed across a wide range of taxa that included the Bacteroidetes, Firmicutes, and Proteobacteria; in contrast, the taxa that were present at higher proportions in the control group were only belonging to Firmicutes (Figure S5B). The relative abundance of the taxa which the absolute amount values exceeded the threshold in both PC2 coefficients and OPLS-DA covariances are shown in the box plots in Figure 3E. These results indicated that microbial memberships and structure differed between the control and AD groups; however, there were no significant differences in alpha diversity scores between the microbiota present in the two dietary groups (Figure S5C). 

Furthermore, to investigate the functions of the microbial community, predicted metagenome profiles were generated by PICRUSt [36] based on the observed 16S rRNA gene sequences. PICRUSt showed that several Kyoto Encyclopedia of Genes and Genomes (KEGG) pathways were significantly different between the control and AD groups (Table S3), although the overall compositions were similar (Figure S6).

### 2.4. The Relationships between Fecal Metabolites and Microbes

**Figure 4:**
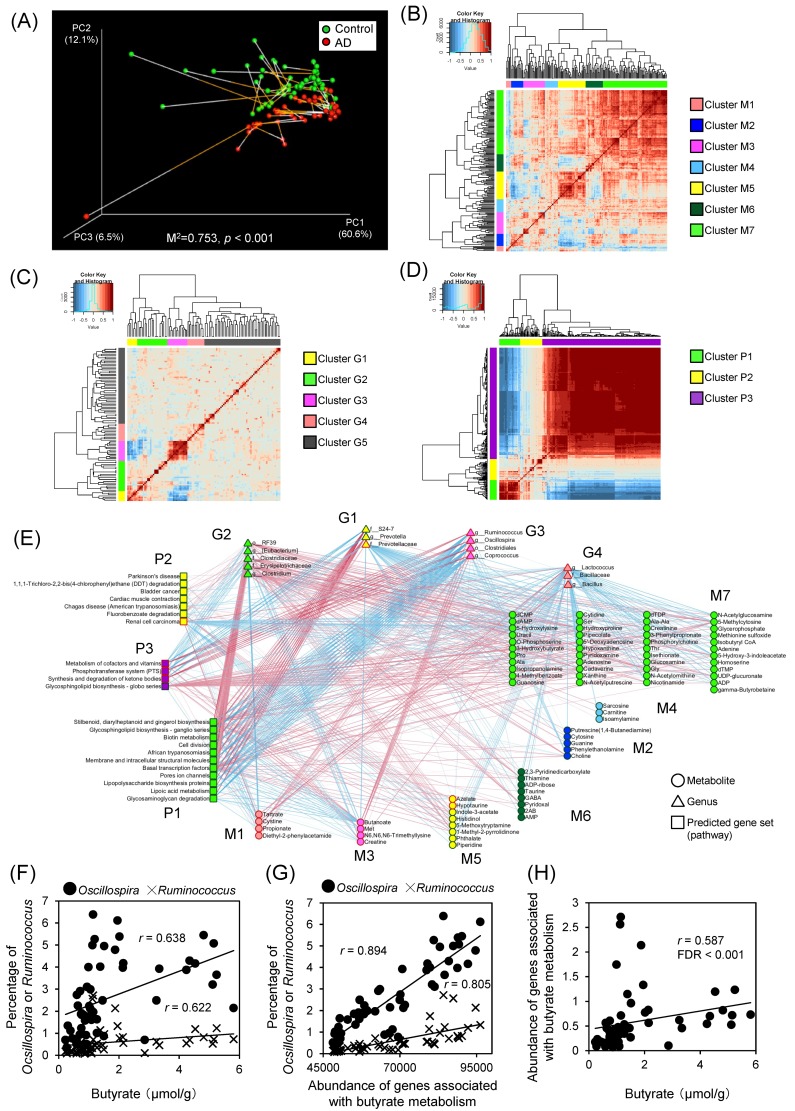
Metabologenomic approach reveals the interactions among abundances of microbial genus, predicted gene set, and metabolite concentration. (**A**) Procrustes analysis combining PCA of intestinal metabolome profiles (end of white line) and weighted UniFrac PCoA of microbiome profiles (end of orange line). The fit of Procrustes transformation over the first three dimensions is reported as the M^2^ value; Autocorrelation maps of (**B**) metabolites, (**C**) bacterial genera, and (**D**) predicted bacterial gene sets (KEGG pathway) based on Spearman’s rank correlation coefficients. Red and blue indicate positive and negative correlation, respectively. Hierarchical clustering based on Euclidean distance was used to separate each metabolite/genus/gene set into clusters shown as side bars (to the right of the respective panels); (**E**) Bacterial genera, predicted gene sets, and metabolites that differed significantly between control and AD were assessed by network analysis. The pairs that yielded significant correlation between each bacterial genus, predicted gene set, and metabolites based on Spearman’s rank correlation coefficients (FDR < 0.05) are portrayed in this network graph. Node shapes denote the type of dataset (circle, metabolites; triangle, genera; square, predicted gene set). Green and red outline colors of nodes denote significantly higher abundance in control or AD group, respectively. Inside color of nodes indicate the clusters defined in Figure 4B–D. Pink and light blue lines denote positive and negative correlation, respectively. Positive correlations (**F**) between relative abundances of *Oscillospira*/*Ruminococcus* and butyrate amount (*r* = 0.638, FDR < 0.001 for *Oscillospira*; *r* = 0.622, FDR < 0.001 for *Ruminococcus*), (**G**) between relative abundance of *Oscillospira*/*Ruminococcus*, and abundance of genes associated with butyrate metabolism (*r* = 0.894, FDR < 0.001 for *Oscillospira*; *r* = 0.805, FDR < 0.001 for *Ruminococcus*), and (**H**) between abundance of genes associated with butyrate metabolism and butyrate concentration (*r* = 0.587, FDR < 0.001).

To clarify the relationship between intestinal metabolome and microbiome profiles in the control and AD groups, Procrustes analysis combining PCA of the metabolome profiles and weighted UniFrac PCoA of the microbiome profiles were conducted to co-visualize the data. Procrustes analyses revealed that plots of both the metabolome and microbiome separated into 2 groups depending on dietary conditions (Figure 4A). This result suggested that both metabolome and microbiome profiles are affected by dietary components, consistent with the results shown in Figure 2B and Figure 3C. Additionally, Procrustes analyses showed the similarity between the metabolome and microbiome plots, suggesting that there were some associations between intestinal microbial structure and metabolome profile. 

We next performed correlation and network analysis to comprehensively understand the crosstalk between microbes and metabolites. To simplify the complex relationships among intestinal microbiome abundance, gene sets, and metabolites, autocorrelation maps and hierarchical clustering analysis (HCA) were used to construct clusters that had the same patterns of changes. The metabolites, genera, and gene sets were clustered into 7 (cluster M1–M7), 5 (cluster G1–G5), and 3 (cluster P1–P3) clusters, respectively (Figure 4B–D and Table S4). Cluster M1 consisted of several metabolites that had higher amounts in AD mice and included molecules such as propionate, tartrate, cysteine, and diethyl-2-phenylacetamide. Cluster M2 included nucleotides (cytosine and guanine), and cluster M4 included methionine metabolism-associated metabolites (sarcosine, carnitine, and methionine sulfone); for both M2 and M4, metabolites were observed at higher amounts in control mice. Metabolites in cluster M3 (butyrate, creatine, Met, and N6, N6, N6-trimethyllysine) were present at higher amounts in AD mice. Cluster M5 included metabolites that were detected at either higher or lower amounts in AD mice. Cluster M6 included amino acids such as Tyr, Phe, Ile, Leu, and Val, all of which were present at higher amounts in control mice. Notably, all branched-chain amino acids (BCAA) fell within cluster M6. Cluster M7 also was composed predominantly of metabolites that were higher amounts in control mice, including amino acids such as Asn, Pro, Ser, Thr, Gln, Gly, Lys, Ala, and Glu. For the genera, clusters G1, G3, and G4 were composed mainly of Bacteroidales, Clostridiales, and Bacilli, respectively. Cluster G2 consisted of 4 different phyla and contained the family Erysipelotrichaceae; the genera belonging to this family tended toward higher abundance in mice fed the control diet. Cluster G5 consisted of 5 different phyla, and included most of the Proteobacteria. For the clusters of PICRUSt-based predicted genes, clusters P1 and P2 primarily consisted of gene sets that exhibited higher abundance in control mice. Cluster P3 constituted a large cluster that included gene sets that were higher abundances in AD mice. 

To obtain more detailed information about the entire interactions between each bacterial taxon, gene set, and metabolite, especially those that differed significantly between the control and AD groups, Spearman’s rank correlation coefficients were calculated, and significantly correlated pairs (FDR < 0.05) were plotted in the form of a network graph (Figure 4E). The genera belonging to cluster G2 tended to show positive correlation with clusters P1, M2, M4, M5, M6, and M7 (containing parameters that were more abundant in control mice), and to show negative correlations with clusters M1, M3, and P3. Similar correlation patterns were observed for the genera in cluster G1 and clusters P1 and P3, however there were a few positive correlations with metabolites. Clusters G3 and G4 consisted predominantly of taxa that were present at higher proportions in the AD group, and these clusters typically exhibited positive correlations with metabolites of cluster M1, and negative correlations with metabolites of clusters M2, M5, and M6 However, clusters G3 and P3 showed positive correlations with M3; in contrast, cluster G4 showed negative correlations with M4 and M7. While a large number of potential pairs yielded significant correlations, we first focused on relationships between amount of choline and relative abundance of bacterial genera. Since the amount of choline was significantly lower in AD compared to control mice. Choline is known to be converted to trimethylamine (TMA) by intestinal microbiota [52] and it has been reported that the order Clostridiales and the genus *Ruminococcus* could be possibly associated with converting choline to TMA [13]. The amount of choline in present study negatively correlated with the abundance of *Coprococcus*, *Lactococcus*, *Oscillospira,* and *Ruminococcus* (Figure S7A), suggesting that these genera may be involved in metabolizing choline to TMA. We next focused on the relationships between the microbiome and butyrate, a metabolite that is known to be produced by intestinal microbiota [32]. In the present work, the amount of butyrate showed significant positive correlation with the relative abundances of bacteria belonging to the genus *Oscillospira* and *Ruminococcus* (Figure 4F). Additionally, the result of PICRUSt showed that predicted abundances of genes associated with butyrate metabolism correlated significantly with the proportion of *Oscillospira* and *Ruminococcus*, and with fecal butyrate concentration (Figure 4G,H). Moreover, there was significant positive correlation between amount of butyrate and predicted gene abundance of butyryl CoA:acetate CoA transferase that converts between butyryl-CoA and butyrate (Figure S7B). It has been reported that some bacterial genera belonging to the Clostridiales, including *Oscillospira*, produce butyrate from dietary fiber [53], suggesting that our metabologenomic approach could be used for the detection of the metabolic activity of intestinal microbiota. 

## 3. Discussion

In this study, the intestinal environments of mice fed control diet or AD were compared by using metabologenomic analysis, our novel approach. Although there were no significant differences observed in body weight between animals maintained on control and AD diets, the results of metabologenomic analyses showed the potentially relations between pairs of metabolites, microbes, and bacterial gene sets. 

CE-TOFMS-based metabolome analysis of murine feces indicated that SCFAs such as butyrate and propionate were higher amounts in the AD group than in the control group. It has been reported that commensal microbes digest dietary fibers in the colon, resulting in the production of SCFAs [22]. In the present study, the amount of fiber in the AD chow was approximately 50% higher than that in the control diet. SCFAs are sensed by GPCRs expressed by host intestinal epithelial cells such as enteroendocrine cells [54]. Propionate is taken up by the liver and used as a substrate for lipogenesis and gluconeogenesis [55]. On the other hand, butyrate induces the differentiation of colonic regulatory T cells and has been reported to exert anti-inflammatory effects [32]. Although mice fed with a high-fat diet have been reported to exhibit decreased fecal SCFA level in mice [56] and rats [57], our findings indicated that animals maintained on AD (which contained higher levels of fat and fiber than the control diet) showed higher fecal amounts of SCFAs. This observation suggested that high fiber intake may facilitate the production of SCFAs even with the ingestion of a high-fat diet. Furthermore, this result suggested that CE-TOFMS-based metabolome analysis may be able to detect the changes in SCFA amount resulting from a mere 1.5-fold difference in the amount of dietary fiber intake.

The results of MSEA and the Mann–Whitney *U* test indicated that the amounts of metabolites associated with methionine metabolism (including AMP, adenosine, *N*,*N*-dimethylglycine, choline, Gly, Ser, homoserine, sarcosine, putrescine, and methionine sulfoxide) were significantly lower, and the amount of Met was significantly higher, in AD mice compared to control mice. Choline that is derived from dietary phosphatidylcholine (e.g., as provided by eggs and meat) is known to be converted to TMA by intestinal microbiota [52] and TMA is further metabolized to trimethylamine-N-oxide (TMAO) by the flavin monooxygenase system in the host liver. Recent studies reported that TMAO is a risk factor for cardiovascular disease [12]. In this study, the amount of choline negatively correlated with the abundance of *Coprococcus*, *Lactococcus*, *Oscillospira* and *Ruminococcus* that were significantly higher in AD-fed mice. It has been reported that the order Clostridiales and the genus *Ruminococcus* were positively correlated with both plasma TMA and TMAO levels in apolipoprotein E knockout mice [13], and several Clostridiales species have been reported to cleave choline [58]. Therefore AD consumption led to increased relative abundance of the genera belonging to Clostridiales such as *Ruminococcus*, and these genera possibly associated with converting choline to TMA. 

PCA and OPLS-DA showed that the concentrations of taurine, thiamine, 3-hydroxybutyrate, and creatinine were significantly lower in AD-fed mice (compared to the control group). Taurine is a semi-essential amino acid and it is known that intestinal microbiota associated with removal of taurine from conjugated bile acids [59]. Additionally, dietary taurine may have anti-inflammatory effects against dextran sulfate sodium-induced colitis in mice [60]. Thiamine, also known as vitamin B1, is produced by some members of the intestinal microbiota such as *Bacteroides thetaiotaomicron*, which can both synthesize and import this compound [61]. It also has been reported that *Acetobacter pomorum* provides thiamine to its host, *Drosophila melanogaster* [62]. 3-Hydroxybutyrate, one of the ketone bodies, has been reported to increase in the serum of mice fed a high-fat/low-carbohydrate ketogenic diet, although the host serum concentration of this compound is unaffected by antibiotic treatment [20]. Therefore, while host levels of this metabolite may be affected by dietary intervention, the effects do not appear to be due to the function of the intestinal microbiota. Creatinine is among the molecules associated with methionine metabolism. It has been reported that the amounts of fecal creatinine and creatine are higher in germ-free mice than in mice colonized with a human intestinal microbiota [38], suggesting that the amounts of intestinal creatinine and creatine may be affected by the intestinal microbiota. 

UniFrac analysis revealed that both the intestinal microbial membership and structure were altered by AD consumption. However, although several previous studies have reported that high-fat or Western diet induces a loss of microbial diversity [63,64,65], AD consumption did not affect the α-diversity of the microbiota in the present study. This apparent conflict may reflect the fact that the AD chow in the present study included a relatively moderate fat content (15.5%) and higher fiber content (5.3%) compared to those of the high-fat diets used in previous studies. According to our microbiome analysis, bacterial taxa belonging to the Clostridiales (including the genera *Oscillospira*, *Ruminococcus*, and *Coprococcus*) were more abundant in the AD group than in the control group. In contrast, unclassified Clostridiaceae were depleted in the AD group compared to the control. These results suggested that bacteria within the same taxonomic order are differentially affected by dietary nutrients. The present work revealed a similar phenomenon for unclassified Prevotellaceae and members of the genus *Prevotella*. Previous studies comparing microbial composition between developed and developing countries reported that high fiber and high carbohydrate consumption yielded elevated proportions of *Prevotella* within the fecal microbiota [66,67]. For example, the intestinal microbiome profiles of Bangladeshi children exhibited high proportions of *Prevotella* and *Oscillospira*, and low proportions of *Bacteroides*, compared to those of American children [66,67]. Similar results were observed for a comparison of the microbial composition of children living in Burkina Faso (Africa) to that of children living in Europe, such that children in Burkina Faso had greater amounts of *Prevotella*, lower amounts of *Bacteroides*, and higher levels of SCFA production. On the other hand, it also has been reported that the proportion of the Prevotellaceae is increased in the feces of obese humans [68]. These results support the hypothesis that various bacteria have different responses to dietary nutrients despite phylogenetic similarities.

In both metabolome and microbiome analysis, there was no significant difference in the distances between samples within the same age and of different ages, although previous studies have reported that the structure of the gut microbiota was affected by host aging [69]. The age of mice used in this study was 8 to 52 weeks old. In contrast, previous studies used 8–24 weeks old mice as the young group, and mice over 18 months as the elderly group [69,70,71,72,73], suggesting that mice aged 52 weeks old might be too young to observe the significant differences on gut environment due to host aging. 

Correlation analyses of integrated metabolome and microbiome datasets reveal large networks within the intestinal environment and numerous potentially related pairs of metabolites, microbes, and gene sets. Autocorrelation maps and hierarchical clustering were used to divide the dataset into clusters that exhibited similar patterns of change, thereby providing a simplified view of the networks and potentially facilitating improved understanding of the complex intestinal ecosystem. According to the network graph, both clusters G3 and G4 consisted of genera that were more abundant in AD mice. Cluster G3 positively correlated with cluster M3 and negatively correlated with cluster M2 and P1, but cluster G4 positively correlated with clusters M1 and M3, and negatively correlated with cluster M2, M4, M5, M6, and M7. These results suggested that these bacteria have different functions, although members of these clusters of organisms had similar responses to the distinct diets. Thus, this analysis potentially supports our understanding of bacterial activity in the intestine, providing information that could not obtained from analysis of only using either metabolome or microbiome. Notably, our results showed that the amount of butyrate exhibits significant positive correlation with the proportions of *Oscillospira* and *Ruminococcus*, an inference that is consistent with a recent study that reported that butyrate is produced primarily by member of the microbial order Clostridiales [32]. Notably, *Oscillospira* have been predicted as a potential butyrate producers based on their genome sequences [53]. Moreover, the results of PICRUSt analysis in the present work showed that abundance of predicted genes associated with butyrate metabolism significantly correlated with the relative abundances of *Oscillospira* and *Ruminococcus*, and with fecal butyrate concentration. Moreover, there was also significant positive correlations between butyrate amount and predicted gene abundance of butyryl CoA:acetate CoA transferase that converts between butyryl-CoA and butyrate. These results demonstrated that our metabologenomic approach has the potential to clarify the activity of bacteria in the intestine based on analyses of metabolome, microbiome, and metagenome datasets. Furthermore, we could integrate a shotgun metagenome dataset from intestinal microbiome instead of predicted metagenome profiles from 16S rRNA-encoding gene sequences. Additionally, for metabolome datasets, other technologies such as liquid chromatography and/or gas chromatography mass spectrometry could be utilized as a complementary tool to obtain in-depth profiles of metabolites that are known to be associated with the function of gut microbiota such as lipids, bile acids, and sugars. Our original integrated approach has the potential to be expanded by including other powerful analytical techniques to yield more detailed and comprehensive information about the intestinal environment. Therefore, the metabologenomic approach is expected to provide new insights into the function of the intestinal microbiota, especially for the investigation of the effects of small alterations in the intestinal environment.

## 4. Materials and Methods 

### 4.1. Animal Experiment

Starting from three weeks of age, male specific-pathogen-free C57BL/6J mice housed at the Central Institute for Experimental Animals (CIEA; Kawasaki, Kanagawa, Japan) were fed *ad libitum* with either a control diet (*n* = 6) or American diet (AD) (*n* = 5). CA-1 chow (CLEA Japan, Inc., Meguro, Tokyo, Japan) was used as the control diet. AD chow was manufactured at CIEA. The nutritional composition of the American diet was defined based on daily human nutritional content in the United States, as described in a report distributed by National Research Council of United States; a diet of equivalent nutritional composition then was formulated as laboratory chow by CIEA [45]. Details of the dietary composition of the control and AD diets are provided in Table S1. Murine fecal samples were obtained from each animal at 8, 12, 24, 36, and 52 weeks of age, and the body weight of each mouse was measured each week during the in-life interval (Figure S2A). Fecal samples were snap-frozen in liquid nitrogen and stored at −80 °C; the resulting specimens were shipped frozen to the Institute for Advanced Biosciences, Keio University, Yamagata, Japan, for metabolome and metagenome analysis. All in-life animal procedures were carried out in accordance with the Institutional Guidelines for Experimental Animal Welfare (Ethics numbers: 09043 (9 October 2009)).

### 4.2. Metabolite Extraction and CE-TOFMS Measurements

Fecal metabolites were extracted from fresh thawed fecal samples (about 10 mg) suspended in 400 μL of 50% methanol in Millli-Q water supplemented with the internal standards (20 μM each of methionine sulfone and D-camphor-10-sulfonic acid (CSA)). The mixture was combined with two 3-mm zirconia beads (BioSpec Products, Bartlesville, OK, USA) and about 100 mg of 0.1-mm zirconia/silica beads (BioSpec Products, Bartlesville, OK, USA) and subjected to 3 min of vigorous shaking using a Micro Smash (TOMY, Nerima, Tokyo, Japan). The suspension was centrifuged at 4600× *g* for 15 min at room temperature, and the resulting supernatant was transferred to a 5-kDa-cutoff filter column (Ultrafree MC-PLHCC 250/pk for Metabolome Analysis, Human Metabolome Technologies, Tsuruoka, Yamagata, Japan). The flow-through was dried under vacuum and the residue then was dissolved in 40 μL of Milli-Q water containing reference compounds (200 μM each of 3-aminopyrrolidine and trimesate). The levels of extracted metabolites were measured in both positive and negative modes by CE-TOFMS as previously described [74]. All CE-TOFMS experiments were performed using an Agilent capillary electrophoresis system (Agilent Technologies, Santa Clara, CA, USA). 

### 4.3. Metabolome Data Processing and Analysis

Raw data were analyzed using our proprietary automatic integration software MasterHands (ver. 2.16.0.15) [74]. Annotation tables were produced from measurements of standard compounds and were aligned with the datasets according to similar *m*/*z* value and normalized migration time. Then, peak areas were normalized against those of the internal standards methionine sulfone and CSA for cationic and anionic metabolites, respectively. Concentrations of each metabolite were calculated based on their relative peak areas and the concentrations of the standard compounds. Metabolites that were detected in at least 4 samples per group were subjected to further data analysis. The amounts of each metabolite were portrayed using a heatmap. HCA was performed by Spearman’s rank correlation. These analyses were performed using Mev TM4 software (ver. 4.8.1; Dana-Farber Cancer Institute) [75]. PCA and orthogonal partial least squares discriminate analysis (OPLS-DA) were run with the SIMCA 15 software (Sartorius Stedim Biotech, Umeå, Sweden) [76,77]. To identify and interpret features of the metabolome profiles, Metabolome Enrichment Set Analysis with Over Representation Analysis mode was conducted using a web-based application [78].

### 4.4. DNA Extraction

Fecal DNA isolation was performed as described previously with some modifications [79]. In short, half to one pellet of murine feces were washed with TE buffer (10 mM Tris-HCl and 1 mM EDTA, pH 8.0). Next, fecal samples were lyophilized for approximately 18 h using a VD-800R lyophilizer (TAITEC, Nagoya, Aichi, Japan). Each freeze-dried fecal sample was combined with four 3.0-mm zirconia beads, approximately 100 mg of 0.1-mm zirconia/silica beads, 400 μL DNA extraction buffer (TE containing 1% (*w*/*v*) sodium dodecyl sulfate), and 400 μL of phenol/chloroform/isoamyl alcohol (25:24:1) and subjected to vigorous shaking (1500 rpm. for 15 min) using a Shake Master (Biomedical Science, Shinjuku, Tokyo, Japan). The resulting emulsion was subjected to centrifugation at 17,800× *g* for 10 min at room temperature, and bacterial genomic DNA was purified from the aqueous phase by a standard phenol/chloroform/isoamyl alcohol protocol. RNA was removed from the sample by RNase A treatment; the resulting DNA sample then was purified again by another round of phenol/chloroform/isoamyl alcohol treatment.

### 4.5. 16S rRNA Gene Sequencing

16S rRNA genes in the fecal DNA samples were analyzed using a MiSeq sequencer (Illumina). The V1-V2 region of the 16S rRNA genes was amplified from the DNA (approximately 10 ng per reaction) isolated from feces using a bacterial universal primer set consisting of primers 27Fmod with an overhang adapter (5′-ACACTCTTTCCCTACACGACGCTCTTCCGATCTAGRGTTTGATYMTGGCTCAG-3′) and 338R with an overhang adapter (5′-GTGACTGGAGTTCAGACGTGTGCTCTTCCGATCTTGCTGCCTCCCGTAGGAGT-3′) [32,80]. PCR was performed with Tks Gflex DNA Polymerase (Takara Bio, Inc., Kusatsu, Shiga, Japan) and amplification via the following program: one cycle of denaturation at 98 °C for 1 min; 20 cycles of amplification at 98 °C for 10 s, 55 °C for 15 s, and 68 °C for 30 s; and a final extension at 68 °C for 3 min. The amplified products were purified using Agencourt AMPure XP kits (Beckman Coulter, Atlanta, GA, USA). The purified products then were further amplified using a primer pair as follows: a forward primer (5′- AATGATACGGCGACCACCGAGATCTACAC-NNNNNNNN-ACACTCTTTCCCTACACGACGC-3′) containing the P5 sequence, a unique 8-bp barcode sequence for each sample (indicated by the string of Ns), and an overhang adapter; and a reverse primer (5′-CAAGCAGAAGACGGCATACGAGAT-NNNNNNNN-GTGACTGGAGTTCAGACGTGTG-3′) containing the P7 sequence, a unique 8-bp barcode sequence for each sample (indicated by the string of Ns), and an overhang adapter. After purification using Agencourt AMPure XP kits, the purified products were mixed in approximately equal molar concentrations to generate a 4 nM library pool after which the final library pool was diluted to 6 pM, including a 10% PhiX Control v3 (Illumina) spike-in for sequencing. Finally, MiSeq sequencing was performed according to the manufacturer’s instructions. In this study, 2 × 300-bp paired-end sequencing was employed.

### 4.6. Analysis of 16S rRNA Gene Sequences Using QIIME

Analysis of 16S rRNA gene sequences was performed as described previously with some modifications [79]. Initially, to assemble the paired-end reads, Fast Length Adjustment of SHort reads (FLASH) (v1.2.11) [81] was used. Assembled reads with an average Q-value < 25 were filtered out using an in-house script. A total of 16,014 filter-passed reads were randomly selected from each sample and used for further analysis. Reads then were processed using the Quantitative Insights into Microbial Ecology (QIIME) pipeline (ver. 1.9.1) [33]. Sequences were clustered into operational taxonomic units (OTUs) based on 97% sequence similarity, and OTUs were assigned to Greengenes Database (ver. 13.8) [82]. Differences in OTU abundance between groups were identified using LEfSe [35].

### 4.7. Metagenome Prediction with PICRUSt

Metagenome prediction with PICRUSt was performed as described previously [36]. A synthetic metagenome was generated based on the observed 16S rRNA gene sequences. First, 16S rRNA gene sequences were clustered into OTUs based on a 97%-similarity threshold and OTUs were assigned to taxonomies based on the Greengenes Database (ver. 13.5) [82]. The resultant OTU table was normalized with inferred 16S rRNA gene copy numbers and predicted microbial metagenomes using a script provided by PICRUSt (ver. 1.0.0) [36].

### 4.8. Metabologenomic Analysis

The scheme of the metabologenomic approach that integrates the metabolome and microbiome analysis is shown in Figure S1. ANOSIM and Procrustes analysis were performed using the QIIME pipeline (ver. 1.9.1). The two-dimensional correlation maps of abundances of genera, of predicted gene sets, and of metabolome abundance based on Spearman’s rank correlation coefficients were drawn using R (gplots package) (https://cran.r-project.org/web/packages/gplots/index.html). The datasets of metabolites, genera, and gene sets were clustered into 7, 5, and 3 clusters (respectively) based on Euclidean distance-based hierarchical clustering. The genera, predicted gene sets, and metabolomes whose abundances differed significantly between the control and AD groups were subjected to further network analysis. The Spearman’s rank correlation coefficients of every pair of abundance of genus, abundance of predicted gene set, and metabolome concentration were calculated using R; pairs that exhibited significant correlation (FDR < 0.05 based on Benjamini-Hochberg correction) were portrayed as a network graph using Cytoscape software (ver. 3.6.1) (http://www.cytoscape.org/) [83]. 

### 4.9. Statistical Analysis 

Non-parametric Mann-Whitney *U* test and FDR values based on Benjamini-Hochberg correction were used for statistical evaluations of comparisons between two groups. Adjusted *p* values (FDR) < 0.05 were considered statistically significant.

### 4.10. Nucleotide Sequence Accession Number 

The microbiome analysis data have been deposited at the DDBJ Sequence Read Archive (http://trace.ddbj.nig.ac.jp/dra/) under accession number DRA007508.

## 5. Conclusions

In conclusion, our metabologenomic analysis, which integrated CE-TOFMS-based metabolome analysis and high-throughput-sequencing-based microbiome analysis, detected changes in the murine intestinal environment associated with AD chow consumption. Correlation analyses of merged metabolome and microbiome information illustrated the existence of numerous networks within the intestinal environment and revealed many potentially related microbe-metabolite pairs. Therefore, the novel metabologenomic approach developed here is expected to facilitate further analyses of the metagenome in intestinal (and other) environments. The metabologenomic approach will be able to provide more detailed information on intestinal metabolites and microbiota, and as well as elucidate new relationships between the two. We hope that the work described here will become a model study that can be used as a standard in the field of multi-omics, enhancing our understanding of intestinal environments.

## Figures and Tables

**Table 1:** Metabolic pathways significantly changed in AD compared to control as assessed by MSEA.

Pathway	Total ^1^	Hits ^2^	Expect ^3^	Fold Change ^4^	*p* Value	Hit Metabolites
Methionine Metabolism	43	11	3.44	3.20	<0.001	AMP, Adenosine, *N*,*N*-Dimethylglycine, Choline, Gly, Ser, Met, Homoserine, Sarcosine, Putrescine, Methionine sulfoxide
Glycine and Serine Metabolism	59	11	4.72	2.33	0.005	AMP, Creatine, *N*,*N*-Dimethylglycine, Gly, Ala, Thr, Ser, Sarcosine, O-Phosphoserine, Met, ADP
Purine Metabolism	74	11	5.93	1.85	0.028	Adenine, AMP, Adenosine, Gly, Guanine, Guanosine, Hypoxanthine, Xanthine, dAMP, ADP-ribose, ADP
Thiamine Metabolism	9	3	0.72	4.16	0.029	AMP, Thiamine, ADP
Alanine Metabolism	17	4	1.36	2.94	0.041	AMP, Gly, Ala, ADP

^1^ Total numbers of metabolites that corresponded in each pathway. ^2^ Observed numbers of metabolites that derived from given dataset in each pathway. ^3^ Expected observed numbers of metabolites that are calculated by given dataset in each pathway. ^4^ Hits/expect.

**Table 2:** Relative abundance of microbial taxa that differ significantly between control and AD mice.

Taxon	Control	AD	*p* Value	FDR	Fold Change
	Mean ± S.D.	Mean ± S.D.			(AD/Control)
Unclassified Erysipelotrichaceae	0.339 ± 0.302	0.042 ± 0.056	0.0020	0.0190	0.13
[Eubacterium]	0.007 ± 0.009	0.002 ± 0.003	0.0022	0.0196	0.24
Unclassified Clostridiaceae	0.681 ± 0.279	0.173 ± 0.109	<0.0001	<0.0001	0.25
Unclassified RF39	0.149 ± 0.074	0.038 ± 0.026	<0.0001	<0.0001	0.26
*Clostridium*	0.055 ± 0.056	0.018 ± 0.013	0.0004	0.0048	0.32
*Prevotella*	2.043 ± 1.070	1.006 ± 0.661	0.0003	0.0037	0.49
Unclassified S24-7	38.452 ± 13.486	27.053 ± 13.126	0.0050	0.0336	0.70
Unclassified Prevotellaceae	3.059 ± 1.890	4.913 ± 2.817	0.0044	0.0336	1.61
*Oscillospira*	1.840 ± 1.258	3.559 ± 1.631	0.0002	0.0026	1.93
Unclassified Clostridiales	0.536 ± 0.409	1.052 ± 0.561	0.0006	0.0066	1.96
*Ruminococcus*	0.389 ± 0.309	0.832 ± 0.728	0.0044	0.0336	2.14
*Coprococcus*	0.221 ± 0.135	0.609 ± 0.305	<0.0001	<0.0001	2.75
Unclassified Bacillaceae	0.001 ± 0.002	0.015 ± 0.031	0.0049	0.0336	23.62
*Bacillus*	0.000 ± 0.000	0.007 ± 0.011	0.0002	0.0026	-
*Lactococcus*	0.000 ± 0.000	0.093 ± 0.086	<0.0001	<0.0001	-

## Supplementary Materials

Supplementary materials can be found at http://www.mdpi.com/1422-0067/19/12/4079/s1.

## References

[1] Sekirov I., Russell S.L., Antunes L.C., Finlay B.B. (2010). Gut microbiota in health and disease. Physiol. Rev..

[2] Arthur J.C., Perez-Chanona E., Muhlbauer M., Tomkovich S., Uronis J.M., Fan T.J., Campbell B.J., Abujamel T., Dogan B., Rogers A.B. (2012). Intestinal inflammation targets cancer-inducing activity of the microbiota. Science.

[3] Belcheva A., Irrazabal T., Robertson S.J., Streutker C., Maughan H., Rubino S., Moriyama E.H., Copeland J.K., Kumar S., Green B. (2014). Gut microbial metabolism drives transformation of MSH2-deficient colon epithelial cells. Cell.

[4] Yoshimoto S., Loo T.M., Atarashi K., Kanda H., Sato S., Oyadomari S., Iwakura Y., Oshima K., Morita H., Hattori M. (2013). Obesity-induced gut microbial metabolite promotes liver cancer through senescence secretome. Nature.

[5] Kau A.L., Ahern P.P., Griffin N.W., Goodman A.L., Gordon J.I. (2011). Human nutrition, the gut microbiome and the immune system. Nature.

[6] Ridaura V.K., Faith J.J., Rey F.E., Cheng J., Duncan A.E., Kau A.L., Griffin N.W., Lombard V., Henrissat B., Bain J.R. (2013). Gut microbiota from twins discordant for obesity modulate metabolism in mice. Science.

[7] Schulz M.D., Atay C., Heringer J., Romrig F.K., Schwitalla S., Aydin B., Ziegler P.K., Varga J., Reindl W., Pommerenke C. (2014). High-fat-diet-mediated dysbiosis promotes intestinal carcinogenesis independently of obesity. Nature.

[8] Qin J., Li Y., Cai Z., Li S., Zhu J., Zhang F., Liang S., Zhang W., Guan Y., Shen D. (2012). A metagenome-wide association study of gut microbiota in type 2 diabetes. Nature.

[9] Le Chatelier E., Nielsen T., Qin J., Prifti E., Hildebrand F., Falony G., Almeida M., Arumugam M., Batto J.M., Kennedy S. (2013). Richness of human gut microbiome correlates with metabolic markers. Nature.

[10] Forslund K., Hildebrand F., Nielsen T., Falony G., Le Chatelier E., Sunagawa S., Prifti E., Vieira-Silva S., Gudmundsdottir V., Pedersen H.K. (2015). Disentangling type 2 diabetes and metformin treatment signatures in the human gut microbiota. Nature.

[11] Koeth R.A., Wang Z., Levison B.S., Buffa J.A., Org E., Sheehy B.T., Britt E.B., Fu X., Wu Y., Li L. (2013). Intestinal microbiota metabolism of L-carnitine, a nutrient in red meat, promotes atherosclerosis. Nat. Med..

[12] Wang Z., Klipfell E., Bennett B.J., Koeth R., Levison B.S., Dugar B., Feldstein A.E., Britt E.B., Fu X., Chung Y.M. (2011). Gut flora metabolism of phosphatidylcholine promotes cardiovascular disease. Nature.

[13] Wang Z., Roberts A.B., Buffa J.A., Levison B.S., Zhu W., Org E., Gu X., Huang Y., Zamanian-Daryoush M., Culley M.K. (2015). Non-lethal Inhibition of Gut Microbial Trimethylamine Production for the Treatment of Atherosclerosis. Cell.

[14] Cahenzli J., Koller Y., Wyss M., Geuking M.B., McCoy K.D. (2013). Intestinal microbial diversity during early-life colonization shapes long-term IgE levels. Cell Host Microbe.

[15] Thorburn A.N., McKenzie C.I., Shen S., Stanley D., Macia L., Mason L.J., Roberts L.K., Wong C.H., Shim R., Robert R. (2015). Evidence that asthma is a developmental origin disease influenced by maternal diet and bacterial metabolites. Nat. Commun..

[16] Hsiao E.Y., McBride S.W., Hsien S., Sharon G., Hyde E.R., McCue T., Codelli J.A., Chow J., Reisman S.E., Petrosino J.F. (2013). Microbiota modulate behavioral and physiological abnormalities associated with neurodevelopmental disorders. Cell.

[17] Kadowaki A., Miyake S., Saga R., Chiba A., Mochizuki H., Yamamura T. (2016). Gut environment-induced intraepithelial autoreactive CD4(+) T cells suppress central nervous system autoimmunity via LAG-3. Nat. Commun..

[18] Buffington S.A., Di Prisco G.V., Auchtung T.A., Ajami N.J., Petrosino J.F., Costa-Mattioli M. (2016). Microbial Reconstitution Reverses Maternal Diet-Induced Social and Synaptic Deficits in Offspring. Cell.

[19] Sampson T.R., Debelius J.W., Thron T., Janssen S., Shastri G.G., Ilhan Z.E., Challis C., Schretter C.E., Rocha S., Gradinaru V. (2016). Gut Microbiota Regulate Motor Deficits and Neuroinflammation in a Model of Parkinson’s Disease. Cell.

[20] Olson C.A., Vuong H.E., Yano J.M., Liang Q.Y., Nusbaum D.J., Hsiao E.Y. (2018). The Gut Microbiota Mediates the Anti-Seizure Effects of the Ketogenic Diet. Cell.

[21] Den Besten G., van Eunen K., Groen A.K., Venema K., Reijngoud D.J., Bakker B.M. (2013). The role of short-chain fatty acids in the interplay between diet, gut microbiota, and host energy metabolism. J. Lipid Res..

[22] Flint H.J., Scott K.P., Louis P., Duncan S.H. (2012). The role of the gut microbiota in nutrition and health. Nat. Rev. Gastroenterol. Hepatol..

[23] Alexeev E.E., Lanis J.M., Kao D.J., Campbell E.L., Kelly C.J., Battista K.D., Gerich M.E., Jenkins B.R., Walk S.T., Kominsky D.J. (2018). Microbiota-Derived Indole Metabolites Promote Human and Murine Intestinal Homeostasis through Regulation of Interleukin-10 Receptor. Am. J. Pathol..

[24] Rothhammer V., Mascanfroni I.D., Bunse L., Takenaka M.C., Kenison J.E., Mayo L., Chao C.C., Patel B., Yan R., Blain M. (2016). Type I interferons and microbial metabolites of tryptophan modulate astrocyte activity and central nervous system inflammation via the aryl hydrocarbon receptor. Nat. Med..

[25] LeBlanc J.G., Milani C., de Giori G.S., Sesma F., van Sinderen D., Ventura M. (2013). Bacteria as vitamin suppliers to their host: A gut microbiota perspective. Curr. Opin. Biotechnol..

[26] LeBlanc J.G., Chain F., Martin R., Bermudez-Humaran L.G., Courau S., Langella P. (2017). Beneficial effects on host energy metabolism of short-chain fatty acids and vitamins produced by commensal and probiotic bacteria. Microb. Cell Fact..

[27] Di Martino M.L., Campilongo R., Casalino M., Micheli G., Colonna B., Prosseda G. (2013). Polyamines: Emerging players in bacteria-host interactions. Int. J. Med. Microbiol..

[28] Kibe R., Kurihara S., Sakai Y., Suzuki H., Ooga T., Sawaki E., Muramatsu K., Nakamura A., Yamashita A., Kitada Y. (2014). Upregulation of colonic luminal polyamines produced by intestinal microbiota delays senescence in mice. Sci. Rep..

[29] Wahlstrom A., Sayin S.I., Marschall H.U., Backhed F. (2016). Intestinal Crosstalk between Bile Acids and Microbiota and Its Impact on Host Metabolism. Cell Metab..

[30] Fukuda S., Toh H., Hase K., Oshima K., Nakanishi Y., Yoshimura K., Tobe T., Clarke J.M., Topping D.L., Suzuki T. (2011). Bifidobacteria can protect from enteropathogenic infection through production of acetate. Nature.

[31] Kimura I., Ozawa K., Inoue D., Imamura T., Kimura K., Maeda T., Terasawa K., Kashihara D., Hirano K., Tani T. (2013). The gut microbiota suppresses insulin-mediated fat accumulation via the short-chain fatty acid receptor GPR43. Nat. Commun..

[32] Furusawa Y., Obata Y., Fukuda S., Endo T.A., Nakato G., Takahashi D., Nakanishi Y., Uetake C., Kato K., Kato T. (2013). Commensal microbe-derived butyrate induces the differentiation of colonic regulatory T cells. Nature.

[33] Caporaso J.G., Kuczynski J., Stombaugh J., Bittinger K., Bushman F.D., Costello E.K., Fierer N., Pena A.G., Goodrich J.K., Gordon J.I. (2010). QIIME allows analysis of high-throughput community sequencing data. Nat. Methods.

[34] Schloss P.D., Westcott S.L., Ryabin T., Hall J.R., Hartmann M., Hollister E.B., Lesniewski R.A., Oakley B.B., Parks D.H., Robinson C.J. (2009). Introducing mothur: Open-source, platform-independent, community-supported software for describing and comparing microbial communities. Appl. Environ. Microbiol..

[35] Segata N., Izard J., Waldron L., Gevers D., Miropolsky L., Garrett W.S., Huttenhower C. (2011). Metagenomic biomarker discovery and explanation. Genome Biol..

[36] Langille M.G., Zaneveld J., Caporaso J.G., McDonald D., Knights D., Reyes J.A., Clemente J.C., Burkepile D.E., Vega Thurber R.L., Knight R. (2013). Predictive functional profiling of microbial communities using 16S rRNA marker gene sequences. Nat. Biotechnol..

[37] Xia J., Psychogios N., Young N., Wishart D.S. (2009). MetaboAnalyst: A web server for metabolomic data analysis and interpretation. Nucleic Acids Res..

[38] Marcobal A., Kashyap P.C., Nelson T.A., Aronov P.A., Donia M.S., Spormann A., Fischbach M.A., Sonnenburg J.L. (2013). A metabolomic view of how the human gut microbiota impacts the host metabolome using humanized and gnotobiotic mice. ISME J..

[39] Yap I.K., Li J.V., Saric J., Martin F.P., Davies H., Wang Y., Wilson I.D., Nicholson J.K., Utzinger J., Marchesi J.R. (2008). Metabonomic and microbiological analysis of the dynamic effect of vancomycin-induced gut microbiota modification in the mouse. J. Proteome Res..

[40] Nagao-Kitamoto H., Shreiner A.B., Gillilland M.G., Kitamoto S., Ishii C., Hirayama A., Kuffa P., El-Zaatari M., Grasberger H., Seekatz A.M. (2016). Functional Characterization of Inflammatory Bowel Disease-Associated Gut Dysbiosis in Gnotobiotic Mice. Cell. Mol. Gastroenterol. Hepatol..

[41] Noecker C., Eng A., Srinivasan S., Theriot C.M., Young V.B., Jansson J.K., Fredricks D.N., Borenstein E. (2016). Metabolic Model-Based Integration of Microbiome Taxonomic and Metabolomic Profiles Elucidates Mechanistic Links between Ecological and Metabolic Variation. mSystems.

[42] Sung J., Kim S., Cabatbat J.J.T., Jang S., Jin Y.S., Jung G.Y., Chia N., Kim P.J. (2017). Global metabolic interaction network of the human gut microbiota for context-specific community-scale analysis. Nat. Commun..

[43] Matsumoto M., Kibe R., Ooga T., Aiba Y., Kurihara S., Sawaki E., Koga Y., Benno Y. (2012). Impact of intestinal microbiota on intestinal luminal metabolome. Sci. Rep..

[44] Kisuse J., La-Ongkham O., Nakphaichit M., Therdtatha P., Momoda R., Tanaka M., Fukuda S., Popluechai S., Kespechara K., Sonomoto K. (2018). Urban Diets Linked to Gut Microbiome and Metabolome Alterations in Children: A Comparative Cross-Sectional Study in Thailand. Front. Microbiol..

[45] Hashimoto H., Arai T., Mori A., Kawai K., Hikishima K., Ohnishi Y., Eto T., Ito M., Hioki K., Suzuki R. (2009). Reconsideration of insulin signals induced by improved laboratory animal diets, Japanese and American diets, in IRS-2 deficient mice. Exp. Clin. Endocrinol. Diabetes Off. J. German Soc. Endocrinol. German Diabetes Assoc..

[46] Turnbaugh P.J., Backhed F., Fulton L., Gordon J.I. (2008). Diet-induced obesity is linked to marked but reversible alterations in the mouse distal gut microbiome. Cell Host Microbe.

[47] Bruce-Keller A.J., Salbaum J.M., Luo M., Blanchard E.t., Taylor C.M., Welsh D.A., Berthoud H.R. (2015). Obese-type gut microbiota induce neurobehavioral changes in the absence of obesity. Biol. Psychiatry.

[48] Kim K.A., Gu W., Lee I.A., Joh E.H., Kim D.H. (2012). High fat diet-induced gut microbiota exacerbates inflammation and obesity in mice via the TLR4 signaling pathway. PLoS ONE.

[49] Bylesjö M., Rantalainen M., Cloarec O., Nicholson J.K., Holmes E., Trygg J. (2006). OPLS discriminant analysis: Combining the strengths of PLS-DA and SIMCA classification. J. Chemom..

[50] Trygg J., Wold S. (2002). Orthogonal projections to latent structures (O-PLS). J. Chemom..

[51] Trygg J. (2002). O2-PLS for qualitative and quantitative analysis in multivariate calibration. J. Chemom..

[52] Vance D.E. (2008). Role of phosphatidylcholine biosynthesis in the regulation of lipoprotein homeostasis. Curr. Opin. Lipidol..

[53] Gophna U., Konikoff T., Nielsen H.B. (2017). Oscillospira and related bacteria–From metagenomic species to metabolic features. Environ. Microbiol..

[54] Nohr M.K., Pedersen M.H., Gille A., Egerod K.L., Engelstoft M.S., Husted A.S., Sichlau R.M., Grunddal K.V., Poulsen S.S., Han S. (2013). GPR41/FFAR3 and GPR43/FFAR2 as cosensors for short-chain fatty acids in enteroendocrine cells vs FFAR3 in enteric neurons and FFAR2 in enteric leukocytes. Endocrinology.

[55] Tremaroli V., Backhed F. (2012). Functional interactions between the gut microbiota and host metabolism. Nature.

[56] Murphy E.F., Cotter P.D., Healy S., Marques T.M., O’Sullivan O., Fouhy F., Clarke S.F., O’Toole P.W., Quigley E.M., Stanton C. (2010). Composition and energy harvesting capacity of the gut microbiota: Relationship to diet, obesity and time in mouse models. Gut.

[57] Jakobsdottir G., Xu J., Molin G., Ahrne S., Nyman M. (2013). High-fat diet reduces the formation of butyrate, but increases succinate, inflammation, liver fat and cholesterol in rats, while dietary fibre counteracts these effects. PLoS ONE.

[58] Moller B., Hippe H., Gottschalk G. (1986). Degradation of various amine compounds by mesophilic clostridia. Arch. Microbiol..

[59] Klaassen C.D., Cui J.Y. (2015). Review: Mechanisms of How the Intestinal Microbiota Alters the Effects of Drugs and Bile Acids. Drug Metab. Dispos. Biol. Fate Chem..

[60] Shimizu M., Zhao Z., Ishimoto Y., Satsu H. (2009). Dietary taurine attenuates dextran sulfate sodium (DSS)-induced experimental colitis in mice. Adv. Experim. Med. Biol..

[61] Costliow Z.A., Degnan P.H. (2017). Thiamine Acquisition Strategies Impact Metabolism and Competition in the Gut Microbe Bacteroides thetaiotaomicron. mSystems.

[62] Sannino D.R., Dobson A.J., Edwards K., Angert E.R., Buchon N. (2018). The Drosophila melanogaster Gut Microbiota Provisions Thiamine to Its Host. mBio.

[63] Thaiss C.A., Itav S., Rothschild D., Meijer M., Levy M., Moresi C., Dohnalova L., Braverman S., Rozin S., Malitsky S. (2016). Persistent microbiome alterations modulate the rate of post-dieting weight regain. Nature.

[64] Sonnenburg E.D., Smits S.A., Tikhonov M., Higginbottom S.K., Wingreen N.S., Sonnenburg J.L. (2016). Diet-induced extinctions in the gut microbiota compound over generations. Nature.

[65] Vangay P., Johnson A.J., Ward T.L., Al-Ghalith G.A., Shields-Cutler R.R., Hillmann B.M., Lucas S.K., Beura L.K., Thompson E.A., Till L.M. (2018). US Immigration Westernizes the Human Gut Microbiome. Cell.

[66] De Filippo C., Cavalieri D., Di Paola M., Ramazzotti M., Poullet J.B., Massart S., Collini S., Pieraccini G., Lionetti P. (2010). Impact of diet in shaping gut microbiota revealed by a comparative study in children from Europe and rural Africa. Proc. Natl. Acad. Sci. USA.

[67] Lin A., Bik E.M., Costello E.K., Dethlefsen L., Haque R., Relman D.A., Singh U. (2013). Distinct distal gut microbiome diversity and composition in healthy children from Bangladesh and the United States. PLoS ONE.

[68] Zhang H., DiBaise J.K., Zuccolo A., Kudrna D., Braidotti M., Yu Y., Parameswaran P., Crowell M.D., Wing R., Rittmann B.E. (2009). Human gut microbiota in obesity and after gastric bypass. Proc. Natl. Acad. Sci. USA.

[69] Spychala M.S., Venna V.R., Jandzinski M., Doran S.J., Durgan D.J., Ganesh B.P., Ajami N.J., Putluri N., Graf J., Bryan R.M. (2018). Age-related changes in the gut microbiota influence systemic inflammation and stroke outcome. Ann. Neurol..

[70] Thevaranjan N., Puchta A., Schulz C., Naidoo A., Szamosi J.C., Verschoor C.P., Loukov D., Schenck L.P., Jury J., Foley K.P. (2017). Age-Associated Microbial Dysbiosis Promotes Intestinal Permeability, Systemic Inflammation, and Macrophage Dysfunction. Cell Host Microbe.

[71] Fransen F., van Beek A.A., Borghuis T., Aidy S.E., Hugenholtz F., van der Gaast-de Jongh C., Savelkoul H.F.J., De Jonge M.I., Boekschoten M.V., Smidt H. (2017). Aged Gut Microbiota Contributes to Systemical Inflammaging after Transfer to Germ-Free Mice. Front. Immunol..

[72] Langille M.G., Meehan C.J., Koenig J.E., Dhanani A.S., Rose R.A., Howlett S.E., Beiko R.G. (2014). Microbial shifts in the aging mouse gut. Microbiome.

[73] Conley M.N., Wong C.P., Duyck K.M., Hord N., Ho E., Sharpton T.J. (2016). Aging and serum MCP-1 are associated with gut microbiome composition in a murine model. PeerJ.

[74] Sugimoto M., Wong D.T., Hirayama A., Soga T., Tomita M. (2010). Capillary electrophoresis mass spectrometry-based saliva metabolomics identified oral, breast and pancreatic cancer-specific profiles. Metab. Off. J. Metab. Soc..

[75] Saeed A.I., Bhagabati N.K., Braisted J.C., Liang W., Sharov V., Howe E.A., Li J., Thiagarajan M., White J.A., Quackenbush J. (2006). TM4 microarray software suite. Methods Enzymol..

[76] Kruger N.J., Troncoso-Ponce M.A., Ratcliffe R.G. (2008). 1H NMR metabolite fingerprinting and metabolomic analysis of perchloric acid extracts from plant tissues. Nat. Protoc..

[77] Wiklund S., Johansson E., Sjostrom L., Mellerowicz E.J., Edlund U., Shockcor J.P., Gottfries J., Moritz T., Trygg J. (2008). Visualization of GC/TOF-MS-based metabolomics data for identification of biochemically interesting compounds using OPLS class models. Analyt. Chem..

[78] Xia J., Wishart D.S. (2010). MSEA: A web-based tool to identify biologically meaningful patterns in quantitative metabolomic data. Nucleic Acids Res..

[79] Murakami S., Goto Y., Ito K., Hayasaka S., Kurihara S., Soga T., Tomita M., Fukuda S. (2015). The Consumption of Bicarbonate-Rich Mineral Water Improves Glycemic Control. Evidence-Based Complement. Altern. Med. ECAM.

[80] Kim S.W., Suda W., Kim S., Oshima K., Fukuda S., Ohno H., Morita H., Hattori M. (2013). Robustness of gut microbiota of healthy adults in response to probiotic intervention revealed by high-throughput pyrosequencing. DNA Res..

[81] Magoc T., Salzberg S.L. (2011). FLASH: Fast length adjustment of short reads to improve genome assemblies. Bioinformatics (Oxford, England).

[82] DeSantis T.Z., Hugenholtz P., Larsen N., Rojas M., Brodie E.L., Keller K., Huber T., Dalevi D., Hu P., Andersen G.L. (2006). Greengenes, a chimera-checked 16S rRNA gene database and workbench compatible with ARB. Appl. Environm. Microbiol..

[83] Shannon P., Markiel A., Ozier O., Baliga N.S., Wang J.T., Ramage D., Amin N., Schwikowski B., Ideker T. (2003). Cytoscape: A software environment for integrated models of biomolecular interaction networks. Genome Res..

