# Supplementary material for: A Metabologenomic Approach Reveals Changes in the Intestinal Environment of Mice Fed on American Diet
Source: Int J Mol Sci. 2018 Dec 17;19(12):4079. doi: 10.3390/ijms19124079 (PMC6321133; doi:10.3390/ijms19124079)
Supplement: Supplementary file 1 [file ijms-19-04079-s001.zip › Supplementary Figures.pdf]

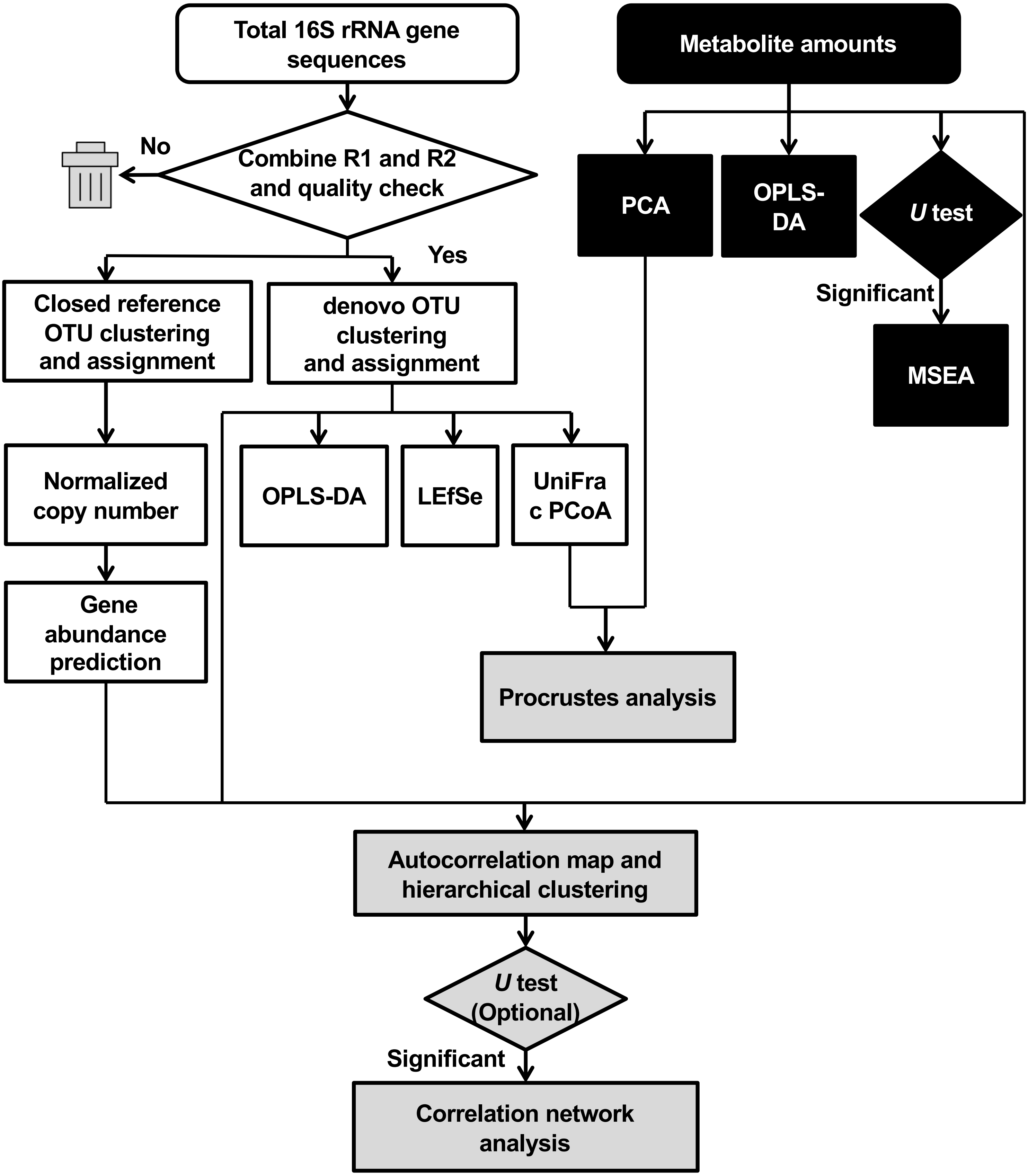


**Figure S1. Computational scheme for the metabologenomic approach.** The entire scheme of the computational analysis used in this study is illustrated as a flowchart. White, black, and gray boxes indicate steps for microbiome, metabolome, and metabologenomic analyses, respectively. OPLS-DA; orthogonal partial least squares discriminate analysis, LEfSe; Linear Discriminant Analysis Effect Size, PCoA; principal coordinate analysis, PCA; principal component analysis, MSEA; metabolite set enrichment analysis, *U* test; Mann-Whitney *U* test.

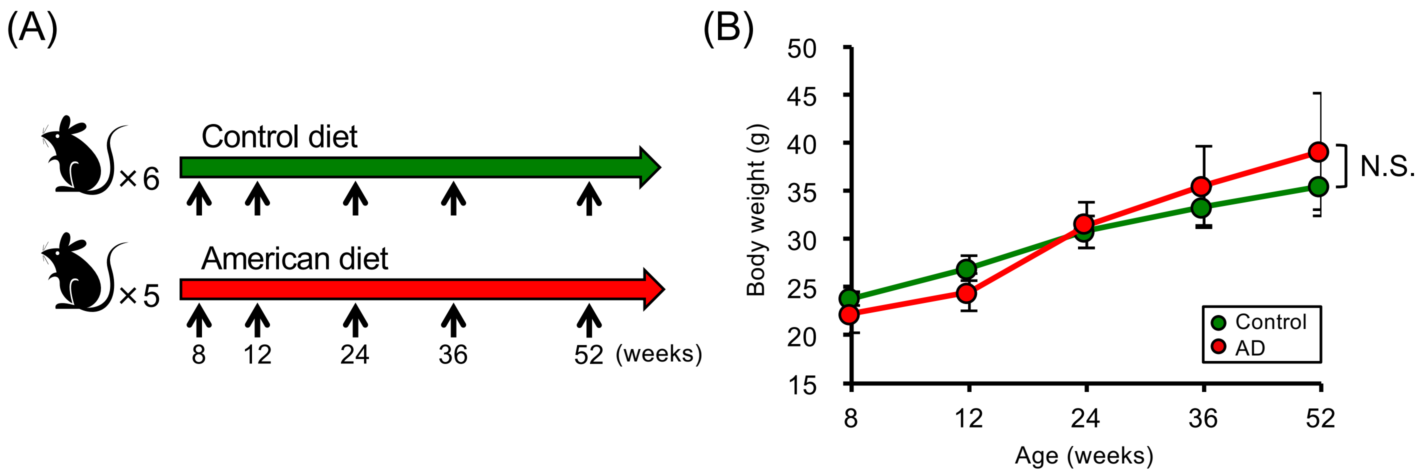


**Figure S2. Murine fecal collection and body weight changes.** (A) Schematic of fecal sampling schedule used in this study. Male specific-pathogen-free C57BL/6J mice were fed *ad libitum* with either a control diet (N = 6) or American diet (AD) (N = 5). Fecal samples were obtained when animals were 8, 12, 24, 36, and 52 weeks old. (B)Body weights of control (N=6) and AD (N=5) mice. In (B), values are plotted as means ± s.e.m. Significance was evaluated by two-tailed repeated-measures ANOVA with Bonferroni post-hoc analysis. N.S., not significant ($p > 0.05$).

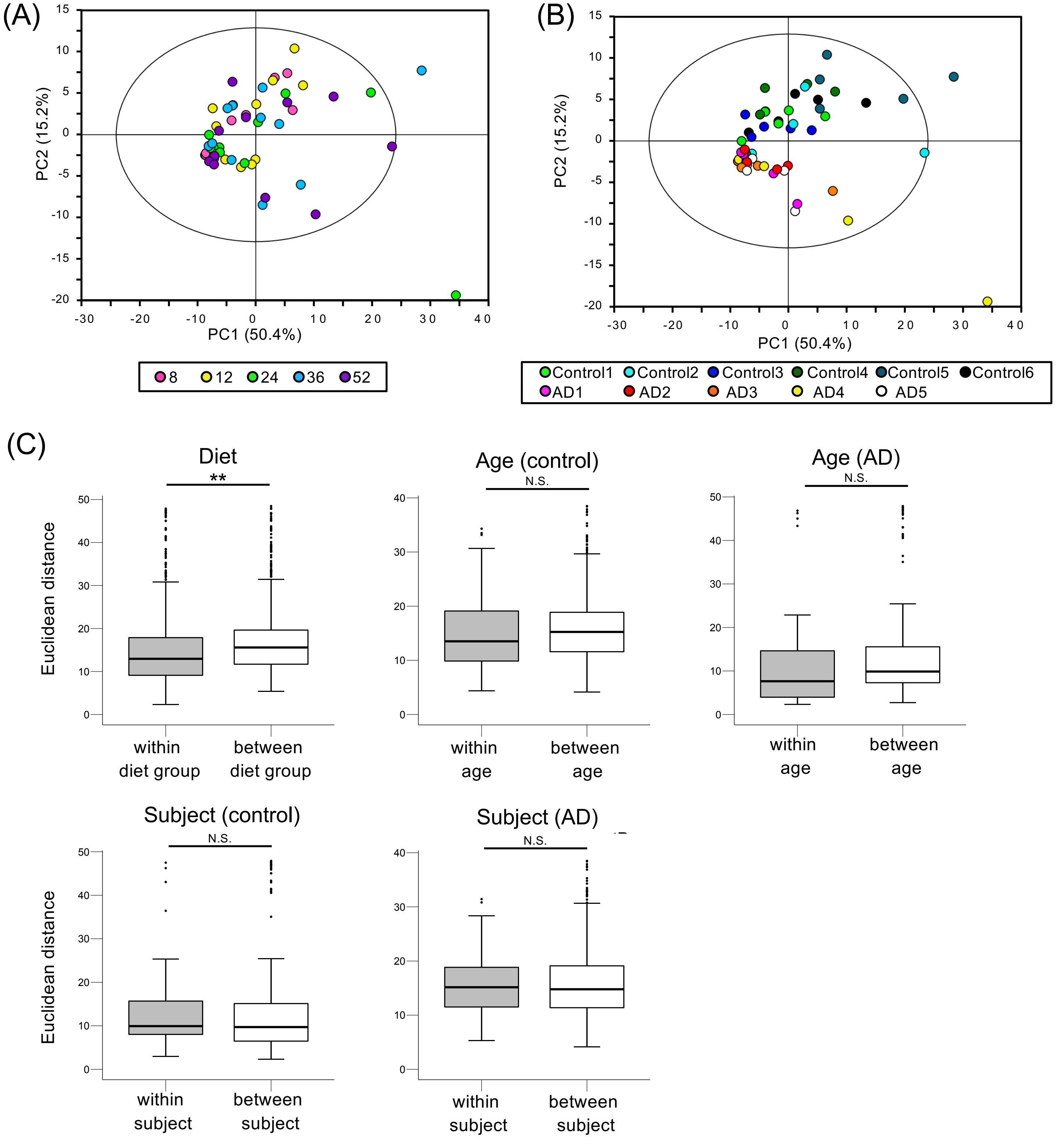


**Figure S3. PCA plots and comparison of Euclidean distances of fecal metabolome profiles.** PCA of the fecal metabolome profiles notarized by Pareto were conducted using SIMCA 15 software (see Methods). The ellipse denotes the 95% significance limit of the model, as defined by Hotelling's t-test. The plot colors indicate (A) age (in weeks) or (B) subjects (mouse nos.); (C) Comparison of metabolome profiles based on Euclidean distances within *vs.* between diet groups, or within *vs.* between samples at different ages, within *vs.* between samples at different subjects. The distances between samples within the same dietary group were significantly shorter than the distances between different dietary groups based on Euclidean distances. However, there was no significant difference in the distances between samples within the same age and of different ages, and distances between samples within the same subject and of different subjects. Significant differences based on Mann–Whitney *U* test are indicated by * $p < 0.05$, ** $p < 0.01$. N.S., not significant ($p > 0.05$).

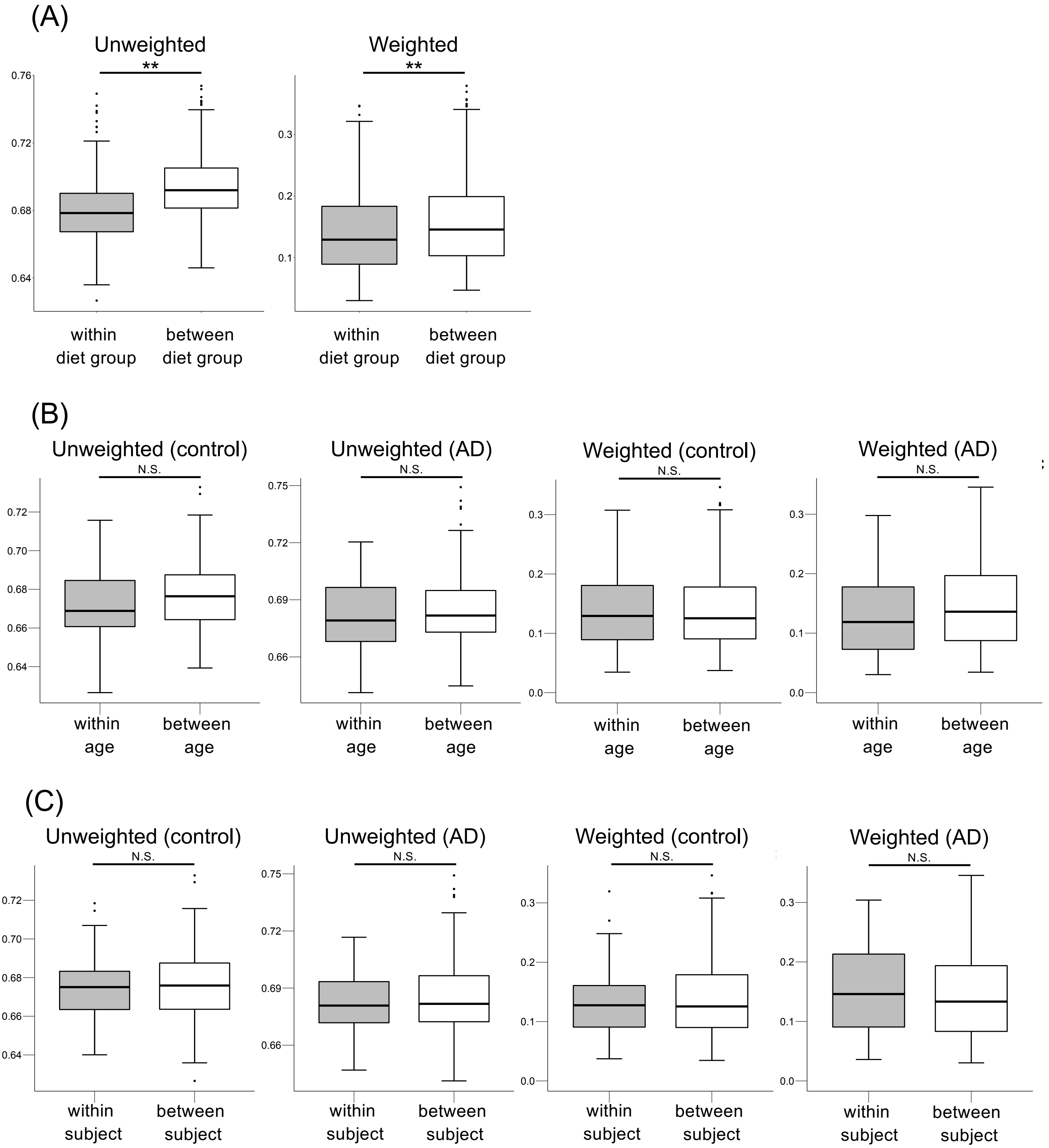


**Figure S4. Comparison of β-diversity of microbiome profiles.** (A) Comparison of microbiome β-diversity based on unweighted or weighted UniFrac distances within *vs.* between diet groups; (B) within *vs.* between samples at different ages; (C) within *vs.* between samples at different subjects. The distances between samples within the same dietary group were significantly shorter than the distances between different dietary groups, based on both unweighted and weighted UniFrac distances. However, there was no significant difference in the distances between samples within the same age and of different ages, and samples within the same subject and of different subjects. Significant differences based on Mann–Whitney *U* test are indicated by * $p < 0.05$, ** $p < 0.01$. N.S., not significant ($p > 0.05$).

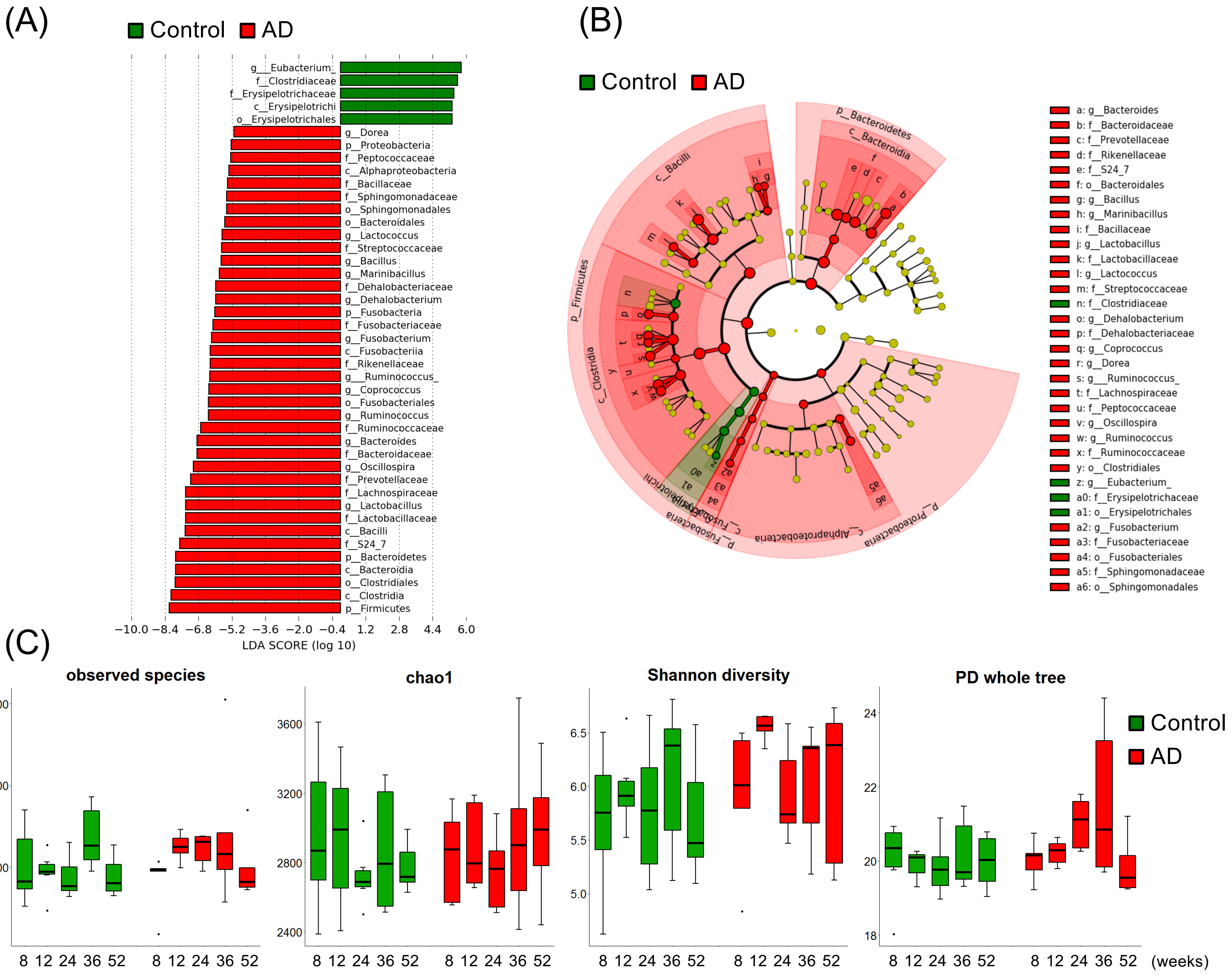


**Figure S5. The results of LEfSe analysis and comparison of $\alpha$-diversity of microbiome profiles.** (A) The control-enriched and AD-enriched taxa (green or red, respectively) exhibited either positive or negative LDA scores, as indicated. The same color coding is used in panels (B); (B) Taxonomic cladogram generated by LEfSe analysis. (C) Comparison of microbiome $\alpha$-diversity based on the number of observed species, chao1, Shannon diversity, and PD whole tree. There were no significant differences between the diet groups based on Mann-Whitney *U* test as assessed by any of these parameters. Significant differences are indicated by * $p < 0.05$, ** $p < 0.01$. N.S., not significant ($p > 0.05$).

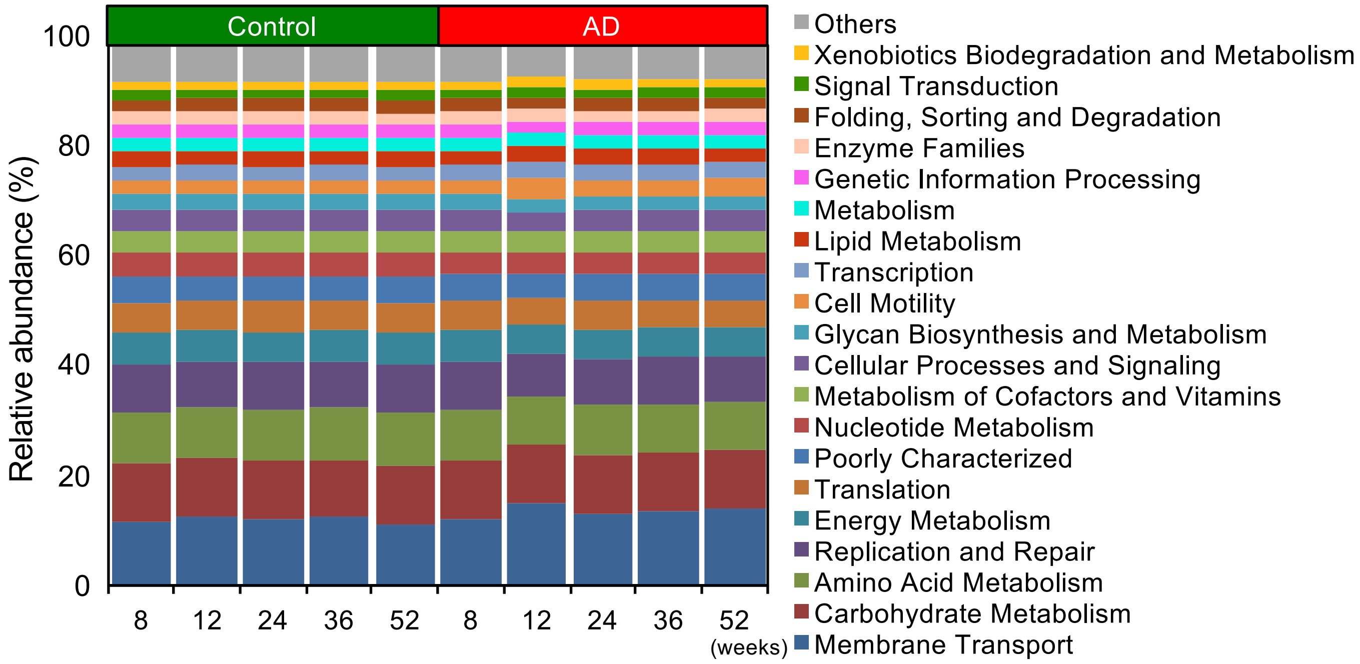


**Figure S6. Predicted metagenome profiles by PICRUSt.** Bar graph showing the relative abundances of KEGG pathways predicted using PICRUSt in control and AD mice.

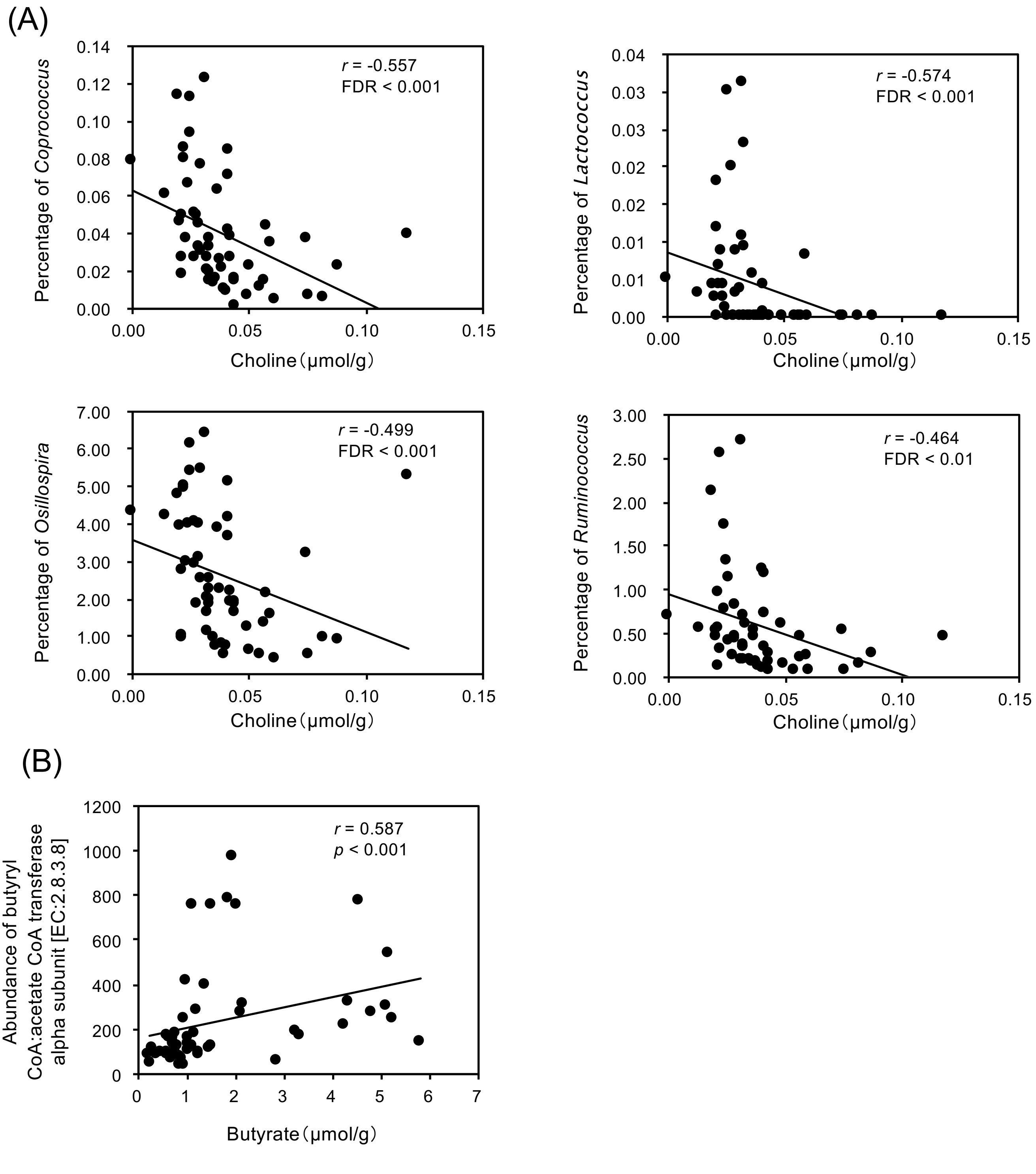


**Figure S7. Correlations between amounts of metabolite, abundances of genus, and predicted gene abundance of enzyme.** (A) Negative correlations between relative abundance of *Coprococcus, Lactococcus, Oscillospira*, or *Ruminococcus*, and choline amount ($r$ = -0.557, FDR < 0.001 for *Coprococcus;* $r$ = -0.574, FDR < 0.001 for *Lactococcus;* $r$ = -0.499, FDR < 0.001 for *Oscillospira*; $r$ = -0.464, FDR < 0.001 for *Ruminococcus*); (B) Positive correlation between predicted abundance of butyryl CoA:acetate CoA transferase alpha subunit and butyrate amount ($r$ = 0.587, $p$ < 0.001).
